# Effects of Cortical Cooling on Sound Processing in Auditory Cortex and Thalamus of Awake Marmosets

**DOI:** 10.3389/fncir.2021.786740

**Published:** 2022-01-05

**Authors:** Marcus Jeschke, Frank W. Ohl, Xiaoqin Wang

**Affiliations:** ^1^Laboratory of Auditory Neurophysiology, Department of Biomedical Engineering, Johns Hopkins University School of Medicine, Baltimore, MD, United States; ^2^Department Systems Physiology of Learning, Leibniz Institute for Neurobiology, Magdeburg, Germany; ^3^Auditory Neuroscience and Optogenetics Group, Cognitive Hearing in Primates Laboratory, German Primate Center-Leibniz Institute for Primate Research, Göttingen, Germany; ^4^Institute of Biology, Otto-von-Guericke-University Magdeburg, Magdeburg, Germany; ^5^Center for Behavioral Brain Sciences (CBBS), Magdeburg, Germany

**Keywords:** auditory cortex, auditory thalamus, cooling, corticofugal feedback, inactivation, spatial processing, temporal modulations

## Abstract

The auditory thalamus is the central nexus of bottom-up connections from the inferior colliculus and top-down connections from auditory cortical areas. While considerable efforts have been made to investigate feedforward processing of sounds in the auditory thalamus (medial geniculate body, MGB) of non-human primates, little is known about the role of corticofugal feedback in the MGB of awake non-human primates. Therefore, we developed a small, repositionable cooling probe to manipulate corticofugal feedback and studied neural responses in both auditory cortex and thalamus to sounds under conditions of normal and reduced cortical temperature. Cooling-induced increases in the width of extracellularly recorded spikes in auditory cortex were observed over the distance of several hundred micrometers away from the cooling probe. Cortical neurons displayed reduction in both spontaneous and stimulus driven firing rates with decreased cortical temperatures. In thalamus, cortical cooling led to increased spontaneous firing and either increased or decreased stimulus driven activity. Furthermore, response tuning to modulation frequencies of temporally modulated sounds and spatial tuning to sound source location could be altered (increased or decreased) by cortical cooling. Specifically, best modulation frequencies of individual MGB neurons could shift either toward higher or lower frequencies based on the vector strength or the firing rate. The tuning of MGB neurons for spatial location could both sharpen or widen. Elevation preference could shift toward higher or lower elevations and azimuth tuning could move toward ipsilateral or contralateral locations. Such bidirectional changes were observed in many parameters which suggests that the auditory thalamus acts as a filter that could be adjusted according to behaviorally driven signals from auditory cortex. Future work will have to delineate the circuit elements responsible for the observed effects.

## Introduction

The thalamus is traditionally conceptualized as a gateway between upstream inputs from subcortical structures to cortical areas (Sherman and Guillery, 2006). In the auditory system the medial geniculate body receives inputs from the inferior colliculus and sends its output to various cortical areas in the primary and secondary auditory cortex (De La Mothe et al., 2006; de la Mothe et al., 2012; Cappe et al., 2009; Saldeitis et al., 2014), but it also receives numerous feedback connections (Rouiller and Durif, 2004; De La Mothe et al., 2006; de la Mothe et al., 2012) that outnumber feedforward connections by far (Deschênes et al., 1998). Together, the feedforward and feedback connections between cortex and thalamus form an intricate cortico-thalamo-cortical loop (Winer and Larue, 1987; Happel et al., 2014; Mukherjee et al., 2020) whose structure-function relationship is still poorly understood (Usrey and Sherman, 2019).

While corticothalamic projections are excitatory, cortical feedback has both excitatory and inhibitory effects on thalamic neurons through disynaptic connections *via* the thalamic reticular nucleus (Cruikshank et al., 2010; Crandall et al., 2015). Diverse functions have been proposed for corticothalamic feedback in the auditory system, including gating of thalamocortical transmission (Yu et al., 2004; Ibrahim et al., 2021), supporting thalamic plasticity during learning (He, 2003; Suga, 2020; Taylor et al., 2021), gain control of cortical input (Saldeitis et al., 2021) and switching dynamics of processing to favor detection or discrimination of stimuli (Guo et al., 2017). Thalamic sensory processing can also be influenced by altering activity further downstream e.g. via further corticofugal projections such as the corticocollicular pathway targeting mostly the non-lemniscal pathway (Winer, 2005; Yudintsev et al., 2021). Thus, more generally, it is apparent that thalamic sensory processing results from the dynamic interplay of feedforward and feedback pathways (Alitto and Usrey, 2003).

Relatively little is known about the role of corticofugal feedback in non-human primates and especially in the auditory system. Although highly successful in rodents, in primates very few studies employed optogenetics to study corticofugal influences up to date (Galvan et al., 2016; Suzuki et al., 2021). This scarcity of studies reflects the technical difficulties associated with implantation of relatively bulky light sources in the area of interest, the required strong light intensities to reach corticofugal output layers 5 and 6 (Yizhar et al., 2011; Dong et al., 2018) as well as the difficulty in translating optogenetic tools from rodents to primates (Jüttner et al., 2019). Another concern particularly relevant for long-term primate experiments is the potential influence on cell physiology due to the introduction and potential overexpression of a foreign protein (Miyashita et al., 2013). In contrast, local cooling is a simple and flexible approach to reversibly inactivate cortical areas and to investigate the role of feedback and feedforward connections of a structure under investigation (Girardin and Martin, 2009; Anderson and Malmierca, 2012; Cooke et al., 2012; Takei et al., 2021). To this end several different designs of cooling devices have been presented so far (Lomber et al., 1999; Cooke et al., 2012). Almost all of these were designed to be implanted and/or affect a larger brain structure or area as a whole. Accordingly, most designs were relatively large with an interface area between cooling device and brain tissue of more than 12 mm^2^. Here, we have developed a small, repositionable cooling device which also allows for simultaneous recording of neural activity directly underneath the probe. The device consists of a small stainless steel foot (~2 mm^2^ footprint) with stainless steel tubing wrapped around via which chilled methanol is pumped to lower the temperature of the device. We used this device in a chronic recording preparation in awake marmosets to study the effect of manipulating corticofugal feedback on cortical and thalamic neural responses to complex sounds that were varied temporally or spatially.

## Materials and Methods

The experiments were conducted at the Johns Hopkins University, Baltimore. All procedures were in accordance with the National Institutes of Health (U.S.) guidelines for the care and use of animals in research. The surgical and experimental techniques were approved by the institutional animal care and use committee of the Johns Hopkins University. Data reported in this article was collected from a total of 4 hemispheres in 3 adult, male common marmosets (*Callithrix jacchus*). Table 1 provides an overview of the number of neurons recorded under the various experimental conditions. The general surgical approach to prepare common marmosets for neurophysiological recordings has been previously reported in detail elsewhere (Lu et al., 2001; Gao and Wang, 2020) and is only briefly described below.

**Table 1:** Overview of single units recorded for the current study split between the various experimental conditions.

	**Left**	**Right**	**Total**
**Total cooling dataset**			
Hemispheres	2	2	4
Cortical units	17	21	38
Thalamic units	74	84	158
**Analysis**	**Condition**	**Number of units**	
**Cortex cooling dataset**			
Waveform distance effects	Baseline + cooled or cooled + recovery	33	
	Baseline/cooled/recovered	21	
Spontaneous and driven activity/distance effects	Baseline + cooled or cooled + recovery	34	
**Analysis**	**Condition**	**Number of units**	
**Thalamus cooling dataset**			
Spontaneous and driven activity/distance effects	Baseline + cooled	144	
Waveform distance effects	Baseline + cooled	142	
	Baseline/cooled/recovered	89	
Frequency tuning	Baseline + cooled	52	
Amplitude modulated sounds	Baseline + cooled	58	
Spatial processing	Baseline + cooled	48	

### Design and Manufacturing of the Repositionable Cooling Probe

**Figure 1:**
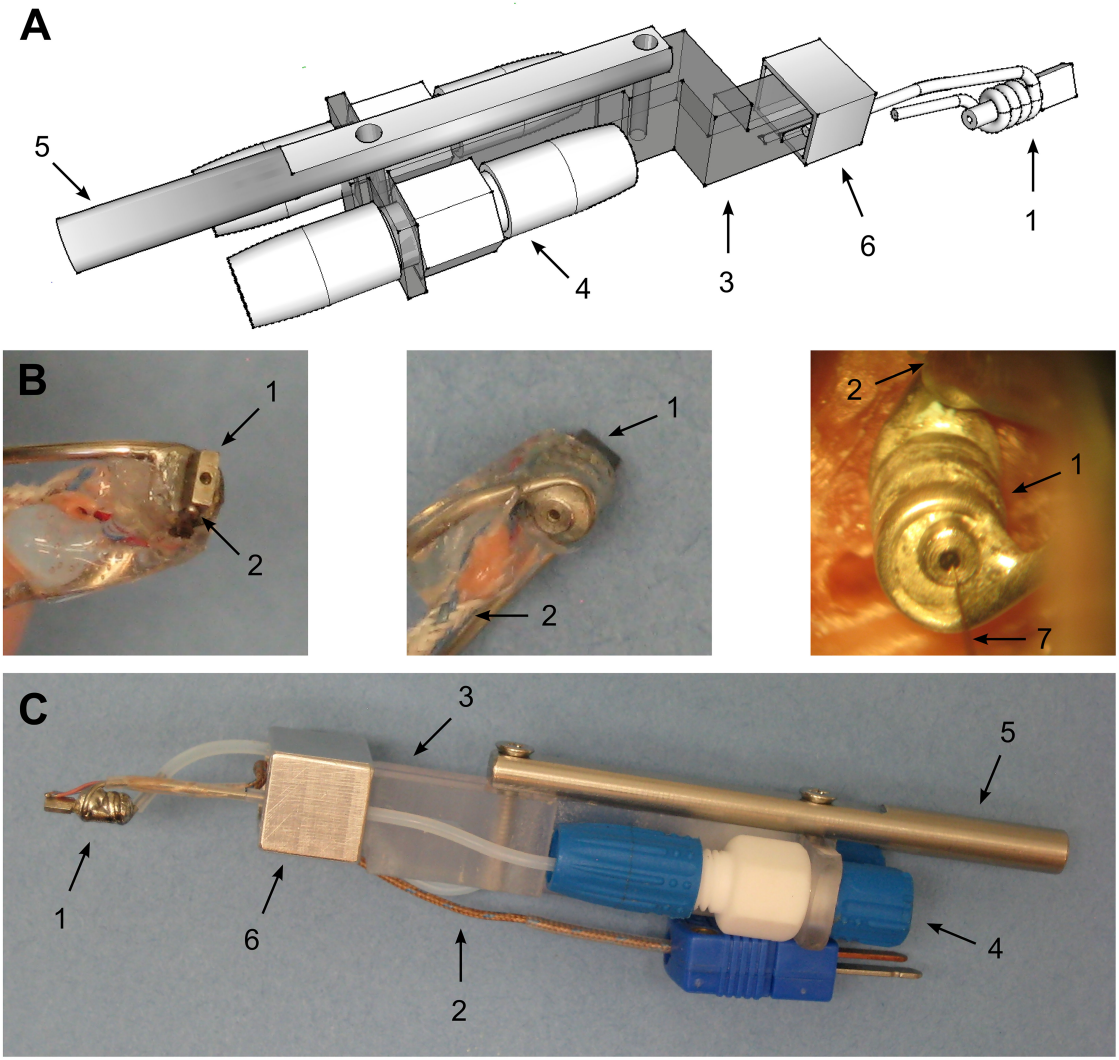
Design of the cooling probe. **(A)** CAD drawing of the developed cooling probe and the holder for use with standard micromanipulators. 1—cooling probe itself consisting of a small stainless steel plate (footsize: 0.95 × 2.5 mm) around whose back a 21G stainless steel tubing was soldered to pump chilled methanol through. 3—plastic holder with an offset front piece to allow for close positioning of recording electrodes. 4—Omnifit® 2-way connector screwed onto the plastic holder to allow the connection of larger diameter cooling fluid supply tubing to smaller tubing leading to the probe itself. 5—Stainless steel adaptor to micromanipulator. Different micromanipulators can be used choosing the appropriate diameter. 6—aluminum clamp to screw the cooling probe onto the probe holder. **(B)** Photographs of the manufactured cooling probe. LEFT – bottom view. Shown is the stainless steel foot with a recording hole drilled to allow access for microelectrodes. This side is touching the dura during experiments. 2 – T-type thermocouple to measure the achieved temperature at the junction of the cooling probe and the dura. MIDDLE – 1 – top view. RIGHT – picture taken during an actual experiment. 7 – single tungsten electrode entering the brain *via* the hole drilled into the cooling probe to record neuronal activity underneath the cooling probe. **(C)** Photograph of the assembled cooling probe and holder. Numbering as in the panels before.

The main foot of the cooling probe was machined from 316 stainless steel rods (McMaster-Carr) using standard milling and lathing techniques. Figure 1 provides an overview of the cooling probe. The back of the foot was lathed to a final diameter of 1.5 mm at a length of ~5–6 mm. The front part of the foot consisted of a plate with dimensions of 0.9 mm width, 2.5 mm length and 3.5 mm height. A small hole was drilled through the center of the foot using a tailstock drill chuck on a lathe and a 0.5 mm drill bit. This enabled recording neuronal activity directly underneath the probe during later experiments. Then, 23 gauge 316 stainless steel tubing (B004UNEY32, Small Parts Inc.) was first wound around a 1 mm stainless steel rod to support tight spiraling around the larger 1.5 mm diameter foot plate shaft and then soldered in place using acid flux and lead free solder. To prevent kinking of the stainless steel tubing while winding, a thinner metal wire was inserted into the stainless steel tube and removed afterwards.

Thermocouples were custom made from T type thermocouple wire (Omega TG-T-30) by soldering the ends of the copper and constantan wires under microscopic control. To achieve this, a small piece of lead free solder was placed on a glass petry dish and molten using a hot stream of air from a heat gun. The thermocouple wire ends were then dipped into the molten solder and the heat gun was turned off to allow the solder to harden leaving a small pellet at the ends of the thermocouple wires (~0.5 mm diameter, see Figure 1). Thermocouples manufactured this way were calibrated at 2 fixed temperature points at 0 and 40°C in a water bath by comparing the measured temperature with a liquid in glass thermometer. These thermocouples were then positioned and glued at bottom of the steel foot and served to monitor the temperature manipulation as well as to ensure proper contact between the brain and the cooling probe.

### Maintenance of Large Scale Craniotomies for Long Term Cooling and Recording

**Figure 2:**
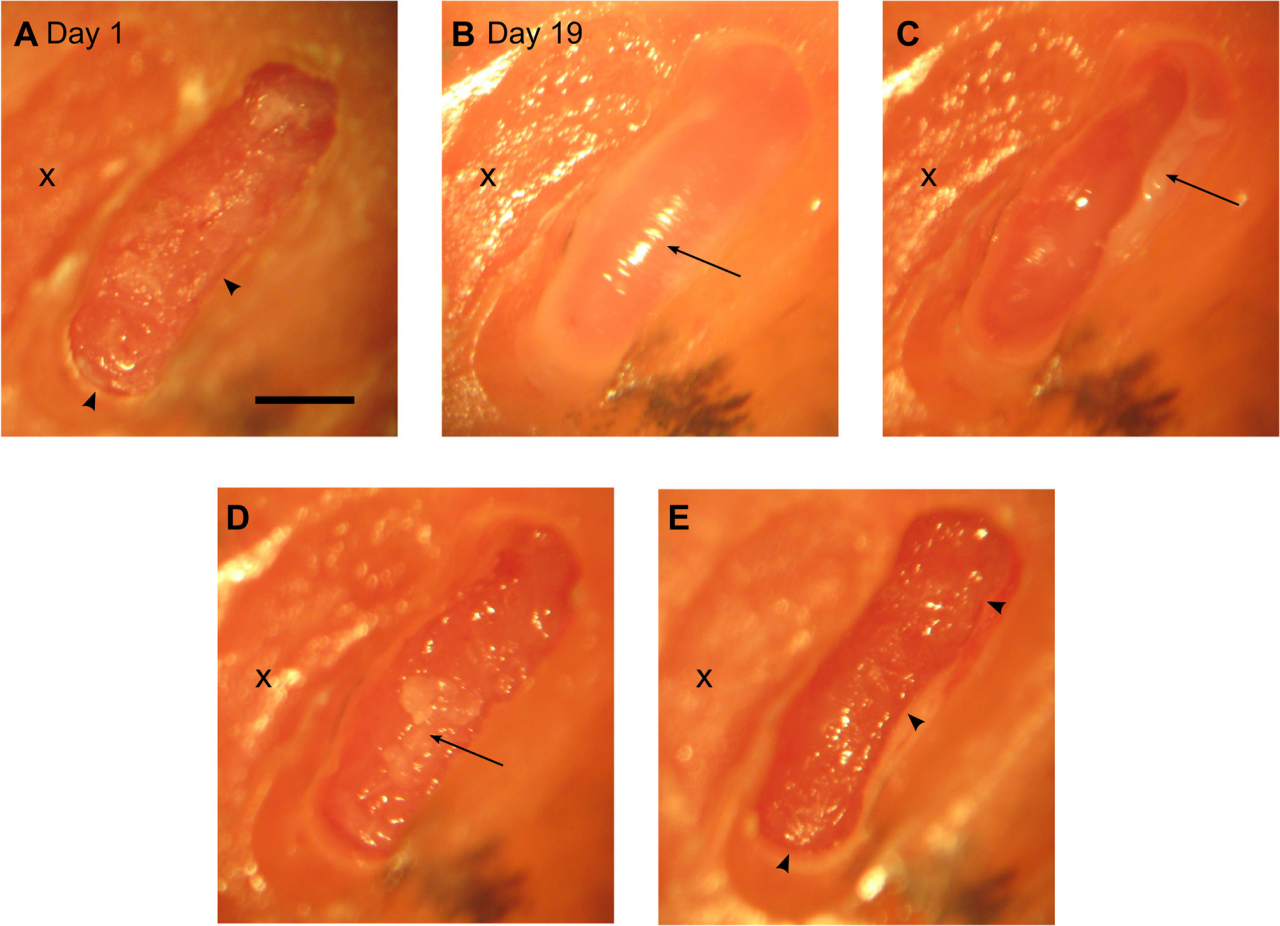
Overview of a progression of a craniotomy to allow access for the cooling probe. **(A)** Day 1: x – old craniotomy. The arrows point to bone edges observed after drilling. Scale bar for all panels = 1 mm. Day 19: the following figures illustrate that it is possible to keep a relatively large craniotomy open for ca. 3 weeks while containing tissue growth. **(B)** – arrow pointing to a layer of tissue on top of the dura. **(C)** – arrow pointing to the tissue pulled aside. **(D)** – arrow pointing to a second layer of tissue about to be removed. **(E)** – final picture after cleaning the tissue again showing the bone edges found at day 1.

To record from marmoset auditory cortex, small craniotomies of ca. 1 mm diameter have commonly been used. From day to day these craniotomies are cleaned and usually closed after 1 to 2 weeks with dental acrylic to perform another craniotomy elsewhere. This approach keeps the dura mater relatively fresh and prevents excessive scarring. In contrast, in the current study craniotomies were substantially larger (1.2 × 3.5–4 mm; Figure 2A) and had to be kept open for several weeks to study cooling effects in the auditory thalamus. Consequently, growth of connective tissue was observed similar to findings during long term recordings in macaques (Figure 2B; see e.g., Spinks et al., 2003). Once a thin layer of connective tissue formed (after 2–3 weeks we estimated a thickness of ca. 200 μm), the craniotomy was thoroughly cleaned and excess tissue removed with hypodermic needles bent into small hooks (26G; Figures 2C–E). This procedure allowed to keep the craniotomy open for several weeks while maintaining a stable distance of the cooling probe placed atop the dura and the cortical elements to be cooled.

### Cooling Procedure

Similar to published work (Lomber et al., 1999; Coomber et al., 2011; Wood et al., 2017; Peel et al., 2020) we opted for a methanol based cooling system were methanol was pumped with an adjustable flow rate (Fluidmetering.com, Q1CSC/QSY (MB) [pump head/pump drive, respectively]; maximum possible flow rate of 92 ml/min) through PTFE tubing (Diba Industries Inc., 008T16-050-20, 008T16-080-20, 008T32-150-10) and the cooling probe. The pump was located outside the recording chamber and placed on foam to reduce vibrations and to eliminate an influence of pumping noise during the experiments. The pump noise was below 45 dB SPL and not measurable inside the double walled recording chamber (attenuation > 47 dB for frequencies higher than 125 Hz; 22 dB SPL noise floor). A styrofoam box with a dry ice bath was used to cool down methanol and was kept on the recording chair ca. 50 cm away from the animals' head. Tubing from the dry ice bath to the cooling probe was insulated with PE foam foil to reduce ice buildup.

The cooling probe was positioned once per day at the beginning of a recording session with a manual stereotaxic micromanipulator (Narishige SM-11) under microscopic control (D.F. Vasconcellos). The probe was advanced until dimpling of the surrounding tissue was observed and then pulled back until dimpling ceased. Due to the small size of the craniotomy relative to the cooling probe it was not always possible to visually verify good contact of the foot plate and dura. In these cases, the cooling probe was advanced until the temperature reading of the thermocouple junction was stabilized above room temperature. Positioning the probe under visual guidance or thermal guidance resulted in similar temperatures recorded at the dura of around 36°C (Omega HH-25TC). During neurophysiological recordings the temperature was monitored and logged to PC with a USB based thermocouple interface (Measurement Computing, USB-2001-TC). For cooling, the pump was set to a nominal flow rate of 10–12% of maximum flow rate. This flow rate led to a temperature of 1–3°C. Once the recorded temperature settled in this range, we recorded single neuron responses to auditory stimuli during a cooled phase. After cessation of cooling by stopping the pump, temperature quickly rose to body temperature and we defined a recovery phase to commence when temperature at the dura reached more than 30°C.

**Figure 3:**
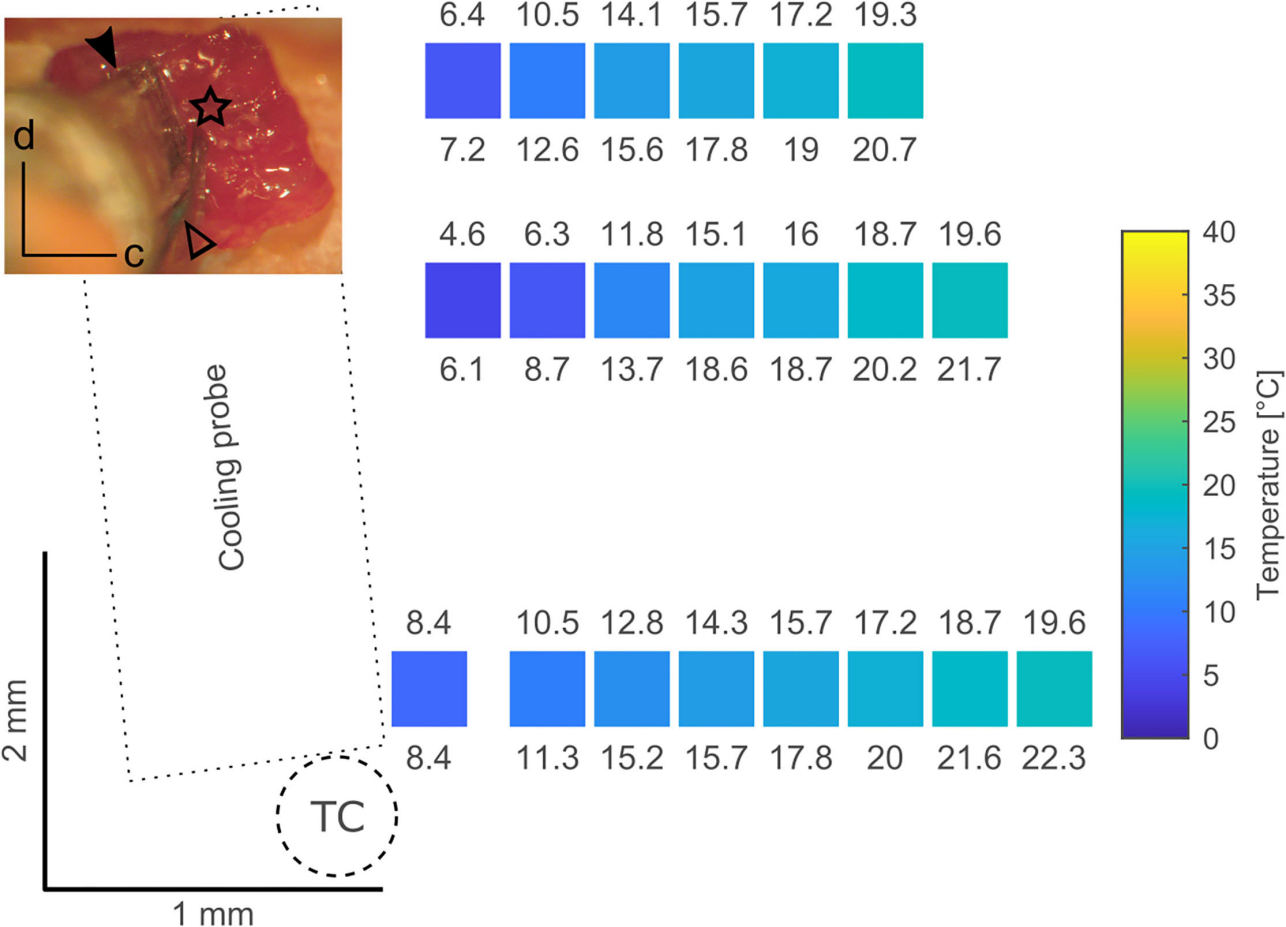
Tests have been conducted using a 3 × 3 mm craniotomy located approximately above area MT in the left hemisphere (inset; d – dorsal, c - caudal). The top part of the craniotomy was overlying the lateral sulcus and thus it's blood vessel. The dimension of the cooling probe (filled arrowhead) in combination with the size of the craniotomy limits the area over which a surface map of temperatures at the dura during cooling can be obtained. In these tests a micromanipulator was used to position a separate microthermocouple (star, located at the thermocouple tip) at several spots over the dura. Coordinates obtained from the micromanipulator during positioning served as a coordinate system to plot the recorded temperatures. All temperatures have been measured after a steady state was achieved (> 10 min) as indicated by the thermocouple (open arrowhead) glued to the cooling probe. In the surface map created from the image in the inset, the position of the colored squares corresponds to the location of a microthermocouple measurement, the color of the squares to the mean temperature and the numbers above and below indicate the minimum and maximum temperature recorded, respectively. Cooling the probe to ca. 5–6°C (measured at the probe thermocouple, TC) results in a temperature of 20°C at a distance of ~1.5 mm thus limiting the cooling effects to the vicinity of the cooling probe. These results are comparable to data presented by Lomber et al. (1999).

We tested the spread of the cooling manipulation across the cortical surface of awake animals. Toward this goal, we opened a 3 × 3 mm large craniotomy located approximately above area MT in the left hemisphere of one animal. A cooling probe was positioned at one edge of the craniotomy and a separate needle style micro-thermocouple was used to measure temperature at various distances to the cooling probe. Our data demonstrate that temperature changes can be observed even several millimeters away from the cooling probe (Figure 3). These findings are qualitatively in line with earlier reports (Coomber et al., 2011), describing a temperature drop to 20–24°C in a radius of 2.5 mm. As large craniotomies were required for further assessment, which would have precluded subsequent long-term evaluation of physiological changes, we did not study temperature gradients in more detail.

### Recording Procedure

**Figure 4:**
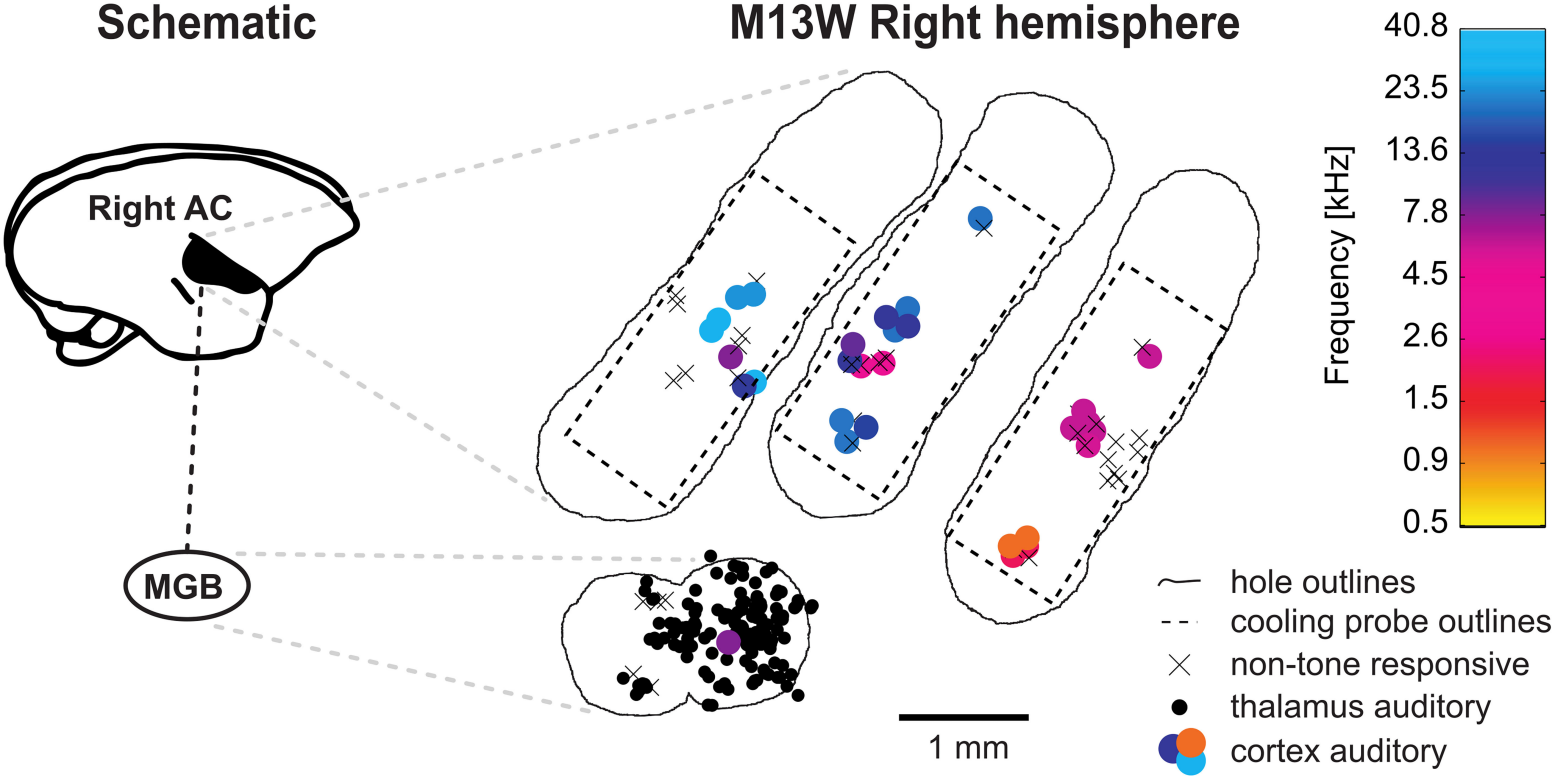
Schematic representation of a typical experiment. Recording from the right hemisphere of animal M13 commonly involved multiple craniotomies (outlined by irregular lines) which were successively created. Usually, one craniotomy for access to the auditory cortex (AC) as well as cooling and a craniotomy for thalamic recordings were open simultaneously. The position of the cooling probe was kept fixed for each craniotomy but the probe was repositioned daily. Through single unit recordings in the auditory cortex field A1 in the core of the auditory cortex was identified by its caudal-to-rostral high-to-low tonotopic gradient. Tone responsive neurons were plotted as filled circles with color depicting their best frequency drawn from a heatmap. Access to the medial geniculate body (MGB) of the thalamus was achieved by offsetting recording holes ventrally at the high frequency region (around 32 kHz) of A1 and inserting recording electrodes at an angle of 60° from vertical. The recording holes for the MGB access are thus located at the lateral belt of the secondary auditory cortex and are, consequently less likely to be tone responsive in cortex.

All experiments were performed in a double-walled soundproof booth (Industrial Acoustics, New York) lined with 3" acoustic foam (Sonex, Illbruck). Animals were tested in daily sessions lasting maximally 6 h but were typically 4.5 h long. We employed the same recording setup as described in Remington and Wang (2019). Briefly, a modified marmoset primate chair was mounted on a pole and acoustic stimulation was performed through one of 16 (covering the upper hemisphere) or 24 (covering a complete sphere) speakers (Fostex dome tweeters, FT28D) located at 1 m from the animal's head. The setup was calibrated to 95 dB SPL at 0 dB attenuation and had a relatively flat frequency response curve (± 3–7 dB) across the frequency range of the presented stimuli. During the experiments, animals were awake, seated in the primate chair and their heads fixed with implanted stainless steel head posts. Single tungsten electrodes (3–12 MΩ; A-M Systems) were employed to either record from the auditory thalamus or the core regions of the auditory cortex, both as described previously (Bendor and Wang, 2005; Bartlett and Wang, 2007). We focused on field AI which we identified by its caudal-to-rostral tonotopic gradient from high to low frequencies. Based on the coarse mapping of field AI the MGB could be approached laterally to AI at an angle of generally 60° from the horizontal plane (Bartlett and Wang, 2007). Entering the brain this way allows to further identify the MGB after passing through a layer of visually responsive neurons (lateral geniculate nucleus). Although we did not attempt to reconstruct electrode tracts, this approach has been shown to lead to the majority of neurons being located in the ventral part of the MGB (Bartlett and Wang, 2011). A hydraulic microdrive (Trent-Wells) mounted on a second manual, stereotaxic micromanipulator (Narishige SM-11) allowed to remotely control the recording depth. In case we studied the effects of cooling cortex on cortical neurons directly underneath the recording probe, the recording electrode was first visually aligned with the axis of the cooling probe and positioned at the top of the cooling probe hole. Then, the electrode was advanced for the height of the cooling probe (measured with a vernier caliper) making sure that the electrode did not bend or make contact with the hole. This electrode position was then defined as 0 μm recording depth. In all other instances the recording depth 0 μm was defined as the position where the electrode made contact with the dura. Where relevant, lateral offsets from the cooling probe were measured from the center of the recording probe and read from the vernier scale of the micromanipulator resulting in an accuracy of 100 μm. For most recording sessions two craniotomies within one hemisphere were used: one for the cortical cooling probe and one for recordings from the ipsilateral auditory thalamus. For an overview of typically recorded data from a hemisphere the reader is referred to the Figure 4.

Under the conditions described here thalamic neurons which are not quickly lost within a few minutes can be held for roughly 45 min. Therefore, we restricted our stimulus sets to be finished within 15–20 min to allow for recording during baseline, cooling, cooled and at least rewarming conditions. Consequently, no attempt was made to characterize the tuning properties of recorded neurons in greater detail and data were only further analyzed if a neuron's response was recorded during baseline, cooled and rewarming phase. Due to the limited time of holding a respective neuron, after cessation of cooling we also did not attempt to recover the tuning properties of the recorded neurons but rather to verify that a unit was not lost during recording in case firing ceased completely during the cooled phase. A neuron was therefore included if we either recovered its response properties and/or could hold a neuron throughout the recording as identified by its waveform. In our hands all waveform related changes were observed to be either gradual allowing to confidently conclude that a single neuron was recorded throughout the procedure or abrupt in which case we did not consider the unit for further analysis. To assess the effects of cortical cooling on thalamic response properties we focused on two types of stimuli: in a subset of neurons we focused on processing of temporal modulations and hereto typically tested neurons with pure tones at various intensities to characterize both frequency (in steps of 1/8^th^ to 1/10^th^ of an octave) and intensity tuning (in steps of 10 dB). At the neurons best frequency (BF, defined as the pure tone frequency eliciting the highest spike rate during stimulus presentation) and 2 levels (one close to threshold and one ca. 30 dB above threshold) or best level [occasionally for non-monotonic neurons; (see Sadagopan and Wang, 2008)] amplitude modulated pure tones were presented spanning several octaves from 2 to 1,024 Hz modulation frequency (modulation depth = 1). Pure tones had a duration of 250 ms while amplitude modulated sounds were presented for at least 500 ms. In another subset of neurons, we studied processing of spatial location. Toward this aim, neurons were typically presented with 250 ms long broadband, frozen noise bursts with a flat frequency spectrum from 2 to 32 kHz at least 10 dB above threshold. The spatial location was systematically varied by changing speaker locations. At the neurons best speaker location (defined as the speaker that led to the highest average firing rate during stimulus presentation), we systematically varied the sound level of the noise burst in steps of 10 dB. All sounds had onset and offset cosine squared ramps lasting 5 ms. The repetition rate of stimuli was slower or equal to 1 Hz.

### Data Analysis

Significantly driven recorded neurons were defined as having at least one stimulus which significantly elevated the firing rate (*t*-test by comparing the spontaneous firing rate with the firing rate during stimulus presentation plus 50 ms; i.e., stimulus related firing). Amplitude modulated sounds were employed to investigate modulation transfer functions (MTF) based on the vector strength revealing stimulus synchronized responses (Goldberg and Brown, 1969; Bartlett and Wang, 2007) or the stimulus related firing rate. To assess statistical significance of synchronized firing the Rayleigh statistic was used (> 13.8 corresponding to a *p* < 0.001). The vector strength based MTF was used to identify the best modulation frequency (BMF vector strength) defined as the modulation frequency leading to the highest significant vector strength and a significant response based on the firing rate. The highest frequency leading to a significant vector strength and significant response was taken as the synchronization boundary (Fcutoff vector strength). Similarly, firing rate based MTFs were analyzed to reveal the best modulation frequency (BMF rate) defined as the modulation frequency leading to the highest significant firing rate. The highest modulation frequency leading to a significant response was taken as the rate boundary (Fcutoff rate). A detailed description of the data analysis of spatial location processing in the auditory thalamus of awake common marmosets is prepared in a separate publication and followed procedures presented in an earlier publication (Remington and Wang, 2019). Briefly, based on the stimulus related firing rate, a spatial receptive field with higher resolution was created by projecting the responses at the various speaker locations onto an array of virtual locations placed on a 5 × 5° grid using a weighted sum of responses at all speaker locations. For graphical representation these responses to virtual locations expressed in a contralateral-to-ipsilateral axis were plotted as a heatmap using a Fournier projection. To express the width of spatial tuning the area of responses at virtual locations with firing rates of at least 50% of the midpoint between the maximum and minimum firing rate was taken and called tuning area (TA). The main tuning vector (centroid) of a given virtual spatial receptive field (essentially the center of mass of the spatial receptive field defined by the firing rate) was calculated with its respective elevation and azimuth. Wilcoxon's signed rank test was used to compare firing between baseline and cooled conditions. A modulation index (MI) was defined as $MI=\frac{rate_{baseline}-rate\text{\_}cooled}{rate_{baseline}+rate\text{\_}cooled}$ to assess the percentage of neurons with changed firing rates (|*MI*| >0.2) between cooling (rate_cooled_) and baseline conditions (rate_baseline_). Pearson correlations were employed to test for a correspondence of changes in one parameter with respect to changes in another. Data were plotted as mean ± STD unless mentioned otherwise. An alpha value of <0.05 was considered to be statistically significant.

## Results

**Figure 5:**
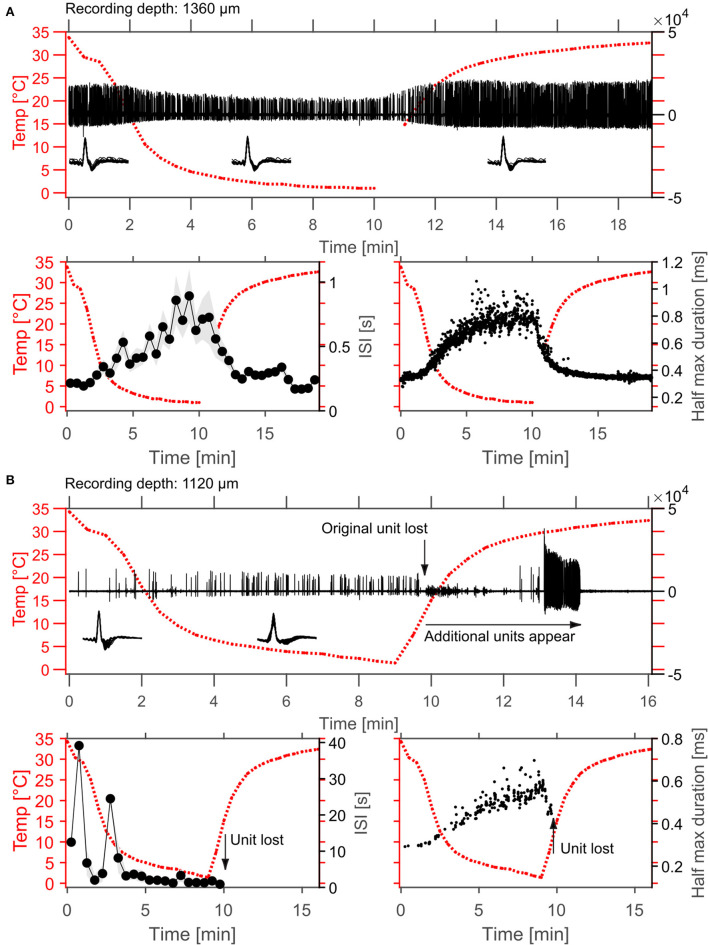
Example effects of cortical cooling on single unit spiking behavior in cortex. **(A)** Spontaneous spiking activity recorded at a depth of 1,360 μm without sound stimulation. In the top panel the high-pass filtered signal is plotted as well as the cortical temperature measured at the dura adjacent to the cooling probe. The isolated single unit waveforms are plotted as insets and sorted according to the different temperatures achieved (before 10°C were reached during cooling, in cooled conditions and after 10°C were reached after cooling). During cooling the firing rate of the unit decreased which was demonstrated by calculation of the average interspike interval (ISI) within 30 s time windows (bottom left panel, plotted as mean ± STD). Interestingly, the recorded unit also changed its' waveform such that amplitude changes were observed during cooling and that the spike width was increased, resulting in an increase of the duration at the half maximum spike amplitude by roughly 2-fold (0.35–0.8 ms). Both, spontaneous firing rate as well as spike widening recovered to baseline levels after cessation of cooling. **(B)** While increases in spike rate during cooling were also observed even at smaller distances to the cooling probe, the waveform changes resulting in widened spike shapes during cooling were consistent (bottom right). Note that the unit depicted in **(B)** was lost during rewarming after ca. 9.5 min. Here, cooling was ceased after 9 min.

In the current study we developed a repositionable cooling probe with small footprint for local cooling and the opportunity for unit recording in close and more distant vicinity of the cooling site (Figure 1). The probe was placed at different locations overlying field AI of the core auditory cortex exposed by craniotomy (Figure 2) to study the effect of manipulating cortical feedback on thalamic processing in the auditory pathway. Experiments were conducted to assess the extent of cooling spread (Figure 3) in awake common marmosets seated in a primate chair. Initial experiments revealed that animals might be able to feel and react to a too rapid decrease in cortical temperature showing discomfort by actively starting to move. Accordingly, flowrate was adjusted such that animals did not show overt reactions to the cooling itself. This precaution allowed for a time constant of ca. 2 min and a stable, steady state temperature of 1–3°C which was reached after 5 min (see Figure 5, a speed for which no overt reaction was observable). Over time we studied the effects of cooling of different parts of field AI by successively opening new craniotomies while closing old craniotomies with bone wax and dental acrylic (Figure 2). Recordings from single neurons in the auditory cortex were used to study the tonotopic organization of the cooled area in order to identify field AI as well as to study direct cooling effects on the physiology of individual cortical cells (Figure 4). In separate recording sessions, the effects of cortical cooling on the physiology of individual thalamic neurons was studied (Figure 4).

### Effects of Cortical Cooling on Single-Unit Responses in the Auditory Cortex

**Figure 6:**
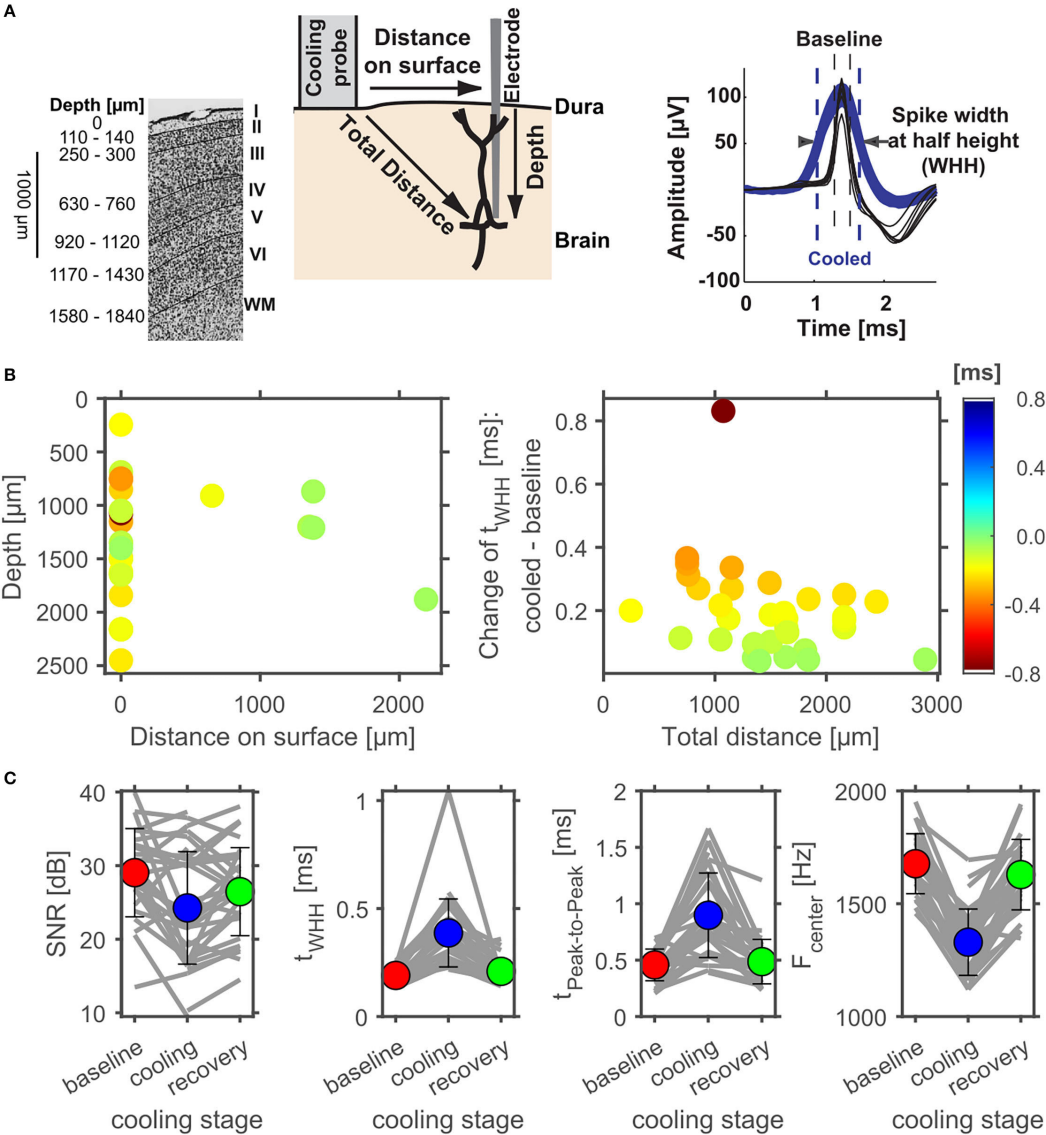
Effect of cortical cooling on spike waveforms at various distances to the cooling probe. **(A)** Schematic representation of the cooling experiment with respect to cortical layers (modified from: Aitkin et al., 1986). Individual layers are identified on the right (WM, white matter) and the estimated average depth of layer transitions are plotted on the left. On the Nissl stain, layer boundaries are indicated by solid black lines. During experiments an electrode was inserted into the brain either through the small hole within the cooling probe itself (see Figure 1B) or at some distance to the cooling probe. Effects of cooling on spike waveforms could therefore be tested at various total distances to the cooling probe. To analyze potential effects of lowering cortical temperature the spike width at half height (WHH) was calculated and compared between different phases of a cooling experiment (right). **(B)** illustrates the change of WHH (color coded) of cortical neurons between the baseline and the cooled condition. Directly underneath the cooling probe only widening of spike waveforms were observed. Next to the probe, waveforms generally did not widen as much. When plotted against the total distance to the cooling probe waveforms widened in a consistent manner with smaller distances leading to larger widening. **(C)** Evaluation of the reversible nature of cooling effects on spike waveforms in cortex. The signal to noise ratio (SNR) did not change consistently due to cooling, while the spike width at half height (WHH) as well as the time between peaks increased during cooling and recovered to a large extent. Conversely, the center frequency of the power spectrum of the spike waveforms decreased during cooling and recovered upon cessation of cooling.

Effects of cortical cooling in awake animals with our custom build probe was tested by investigating spiking properties recorded directly underneath the cooling probe (Figure 5) advancing a single tungsten electrode through the recording hole (see Figure 1B). Based on the available literature (Payne and Lomber, 1999; Malhotra et al., 2004; Nakamoto et al., 2008; Coomber et al., 2011; Wood et al., 2017), we expected single units in the auditory cortex to display a reduction in both, spontaneous and stimulus driven spiking activity upon cooling. For the neuron shown in Figure 5A, recorded at a depth of 1.36 mm underneath the cooling probe, this was indeed observed. The raw data filtered between 0.3 and 6 kHz (Figure 5A top panel) demonstrates the spontaneous activity of a well-isolated single unit waveform which got smaller in amplitude and sparser, indicating a reduction in firing rate. We quantified our observations by calculating the time between successive spikes (inter spike interval, ISI) and observed increases in ISI which depended on the cortical temperature (Figure 5A bottom left). The recorded spike waveform also became wider which we analyzed by calculating the full width at half height of the spike waveforms. This half maximum duration followed the change in temperature closely—almost looking like an inversion of the temperature profile (Figure 5A bottom right). Unlike the expectation stated above not all units in the auditory cortex exhibited decreased firing rates. In contrast, some neurons actually increased their spontaneous firing rate. The unit depicted in Figure 5B was recorded even closer to the cooling probe than the unit in Figure 5A and therefore will have encountered a lower temperature. Still, the ISI decreased substantially from 12.5 s at the beginning of cooling to 2.2 s when 5°C was reached after 5 min. During the same time the spike waveform gradually widened from a half maximum duration of 0.28 s to 0.43 s at 1.8°C. These exemplary data suggest that a consistent effect of local temperature reduction on single neuron physiology could be a widening of spike waveforms. To investigate this further, we pooled data from cortical recordings with cortical cooling and expressed the spike width at half height (WHH) as a function of distance from the cooling probe (Figure 6A). Together, the data demonstrate that the spike waveform of all investigated single units widened to various degrees during cooling (Figure 6B). Directly underneath the cooling probe only widening of spike waveforms was observed. Further, we observed negative correlations for all changes in measures of spike waveforms and the recording depth (Pearson correlation; WHH: Rho = −0.37, *p* = 0.037; time from peak-to-peak: Rho = −0.3, *p* = 0.036; center frequency: Rho = 0.448, *p* = 0.01). Next to the probe, waveforms generally did not widen as much. When plotted against the total distance to the cooling probe waveforms widened in a consistent manner with smaller distances leading to larger widening (Pearson correlation; WHH: Rho = −0.33, *p* = 0.06; time from peak to peak: Rho = −0.366; *p* = 0.039). These results are compatible with a temperature gradient achieved during cooling spreading from the cooling probe itself. Changes in spike waveforms were also reversible. All investigated spike measures including signal-to-noise ratio (SNR), the WHH, the time from peak to peak as well as the center frequency were indistinguishable during baseline and recovery conditions (Figure 6C), while cooling increased the WHH, the time from peak-to-peak and decreased the center frequency (repeated measures ANOVAs with factor cooling stage; WHH: *F*_(2, 74)_ = 33.8, *p* = 3.67e-11; time from peak-to-peak: *F*_(2, 74)_ = 32.3, *p* = 8.11e-11; center frequency: *F*_(2, 74)_ = 61.9, *p* = 2.85e-12). The SNR changed very little during cooling suggesting, on average, a stable recording quality throughout the experiment (repeated measures ANOVA with factor cooling stage *F*_(2, 74)_ = 6.49, *p* = 0.00693; no significant *post-hoc* test).

### Effects of Cortical Cooling on Spontaneous and Stimulus Driven Activity in the Auditory Cortex

**Figure 7:**
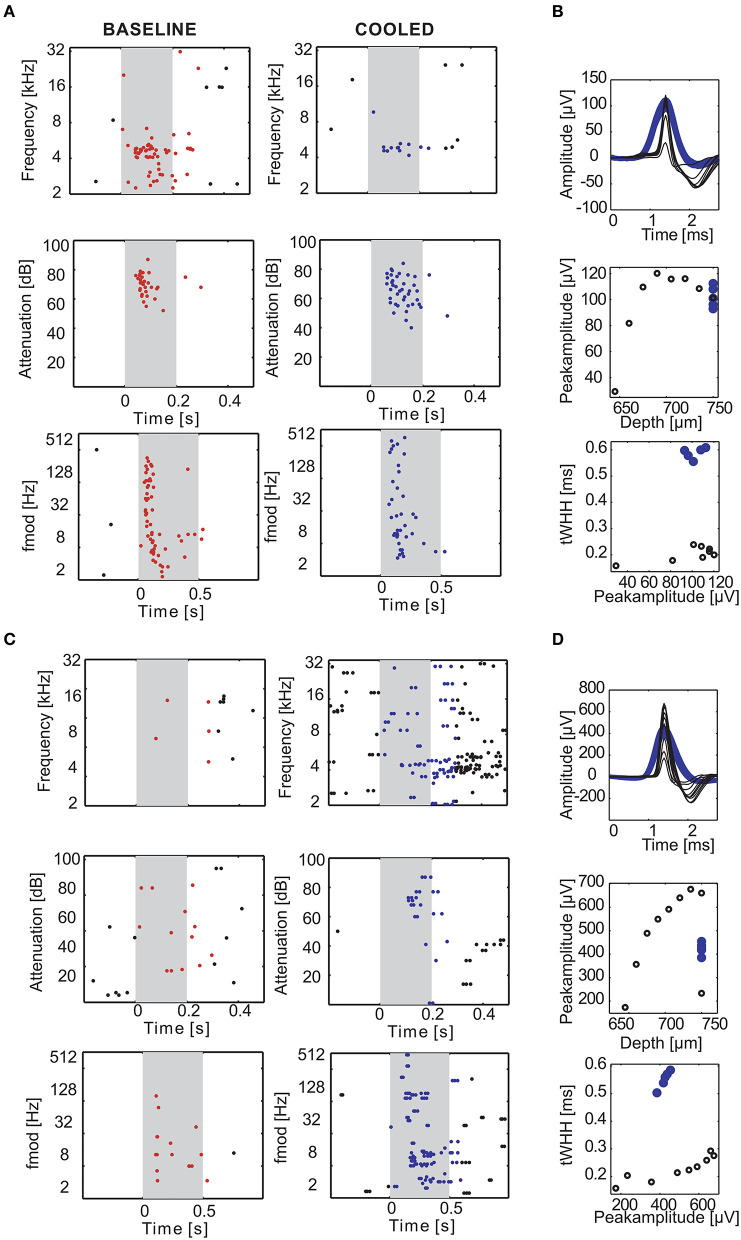
Examples of cortical cooling effects on single units in the auditory cortex. During cortical cooling response properties of cortical neurons can change in diverse ways. The rasterplots in **(A,B)** illustrate an experiment in which 2 neurons were recorded simultaneously at the same electrode 750 μm directly underneath the cooling probe. The gray shaded area corresponds to the stimulus duration. Stimulus related firing was evaluated based on a time window which included the stimulus duration plus 50 ms. Spikes that contributed to this stimulus related firing were plotted in red during baseline and blue during cooling. While the single unit in **(A)** was driven by pure tones around 4.6 kHz (top) at 10–20 dB SPL (middle) and various amplitude modulation frequencies (bottom) the second single unit was not **(B)**. Cortical cooling led to reduced firing rates in **(A)** without changing the overall tuning properties of the neuron. In contrast, cooling increased firing rates in **(B)** and revealed frequency tuning to around 4 kHz. Note that, since the neuron in **(B)** was not driven during the baseline condition the stimulus sets were only tailored toward neuron A. Even though the effects of cortical cooling on tuning properties of the two neurons were different, the waveforms of both neurons widened **(C,D)**. Average waveforms for different stimulus sets during baseline condition were plotted in black and blue for cooled condition. The waveform changes cannot be explained by undeliberate movements of the electrode as purposeful movements resulted in different peak amplitudes without significant changes in spike width **(C,D)**.

Next, we investigated how processing of sounds is changed locally due to cooling cortex. Figure 7 illustrates two representative single units recorded concomitantly in a depth of 750 μm. Under baseline conditions, the unit in panel A displayed a non-monotonic response function to pure tones around 4.6 kHz at sound pressure levels from 10 to 20 dB SPL and also exhibited a phasic response to sinusoidal amplitude modulated tones (sAM) with modulation frequencies between 2 and 128 Hz, while the unit in panel B was not driven by these stimuli. In a cooled state the driven firing rate of the unit in panel A decreased and the response pattern was prolonged. In contrast, the unit in panel B now displayed an offset response to pure tones around 4 kHz and had a threshold for 4.6 kHz (the best frequency identified for the unit in A) around 10–20 dB SPL. Further, the unit now responded to amplitude modulated tones. These observations suggest that the effect of cortical cooling on local processing of sounds could be diverse. When analyzing the spike waveforms for both units (Figures 7B,D) a substantial widening was observed in each case. A potential alternative explanation to spike waveform changes than a local reduction in temperature would be relative movements of the recording electrode and the recorded neuron (Gold et al., 2006) which could be due to shrinkage or expansion of the cooled tissue. During the recovery phase after cooling was ceased we therefore deliberately moved the recording electrode. As expected, the amplitude of the spike waveform changed with varying electrode depth. While electrode movements resulted in instances where the spike waveform amplitudes were comparable between the baseline and the cooled state (unit in Figure 7B had a peak amplitude of 80–120 μV and in a depth of 675–750 μm during cooled state and baseline; Figure 7D: peak amplitude of 350–500 μV in the cooled state and in a depth of 660–675 μm during recovery), the WHH calculated was about 3 times longer in the cooled state (Figure 7B: 0.2 ms during baseline and ca. 0.6 ms in a cooled state; Figure 7D: 0.2 ms during baseline and ca. 0.6 ms in a cooled state) and never overlapped between both states. This suggests that changes in spike waveform are a direct consequence of cooling and are in line with earlier reports in anesthetized cats (Girardin and Martin, 2009).

**Figure 8:**
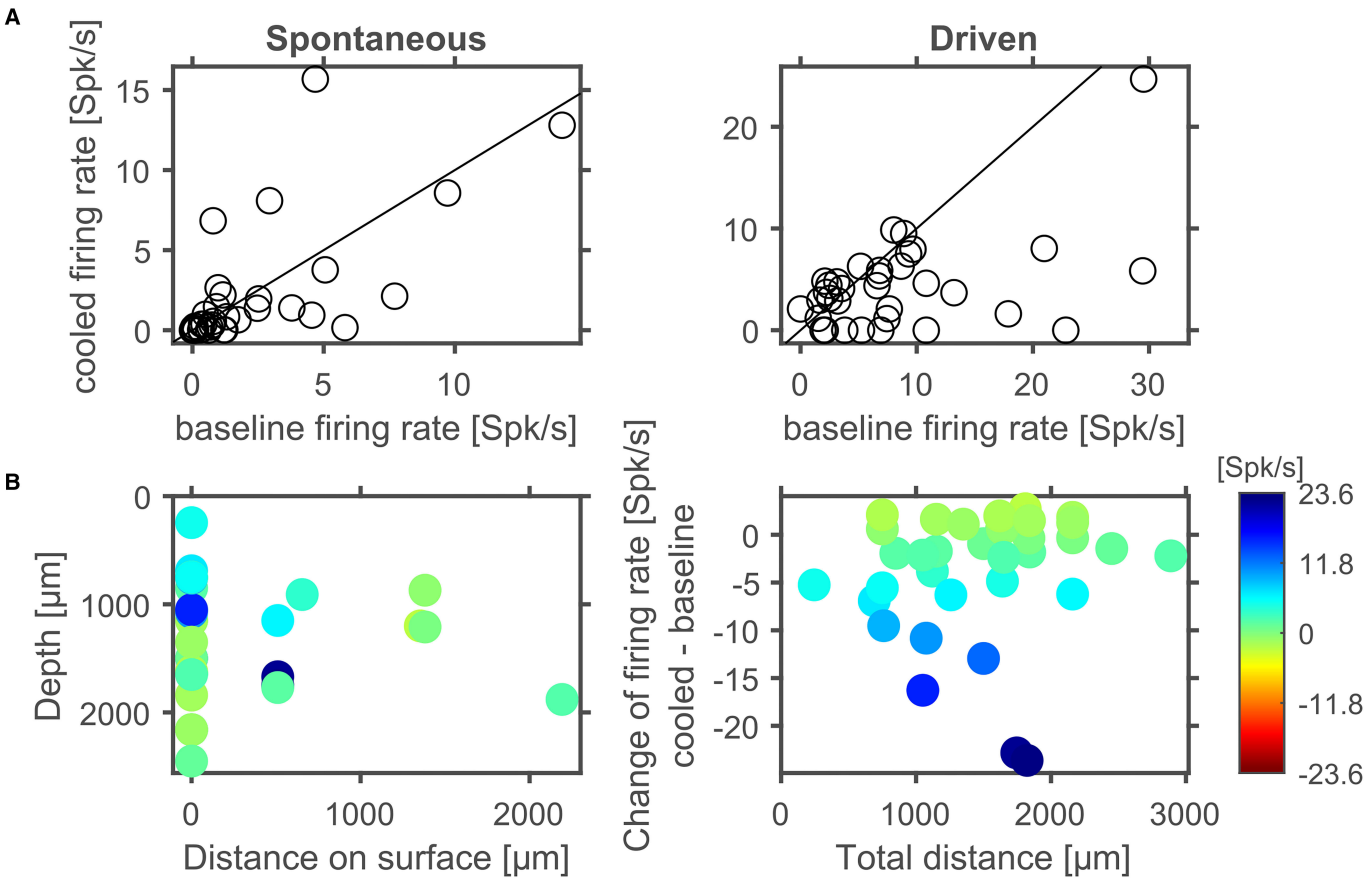
In cortex spontaneous and driven firing rates were effected by cortical cooling. **(A)** Cortical cooling leads mostly to decreased, but also increased, spontaneous and driven firing rates in cortex. Across the ensemble of neurons tested the stimulus driven activity was significantly reduced while the change of spontaneous activity showed a trend for reduced firing rates. Changes in stimulus driven activity did not show a clear correlation with distance indicating that the effects of cortical cooling spread over several millimeters **(B)**.

Next, we separately analyzed spontaneous and stimulus driven firing rates across the sample of units studied. As suggested from the individual examples illustrated above, decreased, but also increased, spontaneous and driven firing rates were found due to cortical cooling. Overall, we observed a significant reduction of driven firing rates (Wilcoxon signed rank test, *Z* = −3,103, *p* = 0.0019, 44% of neurons decreased [with a modulation index (MI) > 0.2] while 18% increased their firing rate [MI < −0.2]; Figure 8A right) and a trend for reduced spontaneous firing rates (Wilcoxon signed rank test, *Z* = −1,769, *p* = 0.0768; 47% of neurons decreased [MI > 0.2] while 32% increased their firing rate [MI < −0.2]; Figure 8A left). Many units stopped responding to sound stimulation in the cooled state. For units with an increase in driven firing rates the increase was comparatively small. The lack of a clear reduction of spontaneous firing rates might be due to the overall low spontaneous firing rate of auditory cortical neurons. For units that were recorded directly underneath the cooling probe, we observed decreased spike rates that expanded to a depth of at least until 2.5 mm. When plotting the observed changes in driven firing rate against the distance of the recorded unit to the cooling probe (Figure 8B), no dependence of the change in firing rate between the baseline and cooled state and the distance was observed (Pearson correlation; recording depth: Rho = 0.052, *p* = 0.77; total distance: Rho = 0.114, *p* = 0.52). This finding held when the analysis was focused only on units directly underneath the cooling probe. Here, neither the change in spontaneous activity nor the change in driven firing rate displayed a correlation with recording depth (Pearson correlation: spontaneous activity: Rho = 0.080615; *p* = 0.70167; driven activity: Rho = 0.29927; *p* = 0.14614). Together, these data suggest that cortical cooling affects sound processing locally and exerts an influence which extends for several millimeters including the corticothalamic output layers 5 and 6 in marmoset field AI (Figure 6A).

### Effects of Cortical Cooling on Spontaneous and Stimulus Driven Activity in the MGB

**Figure 9:**
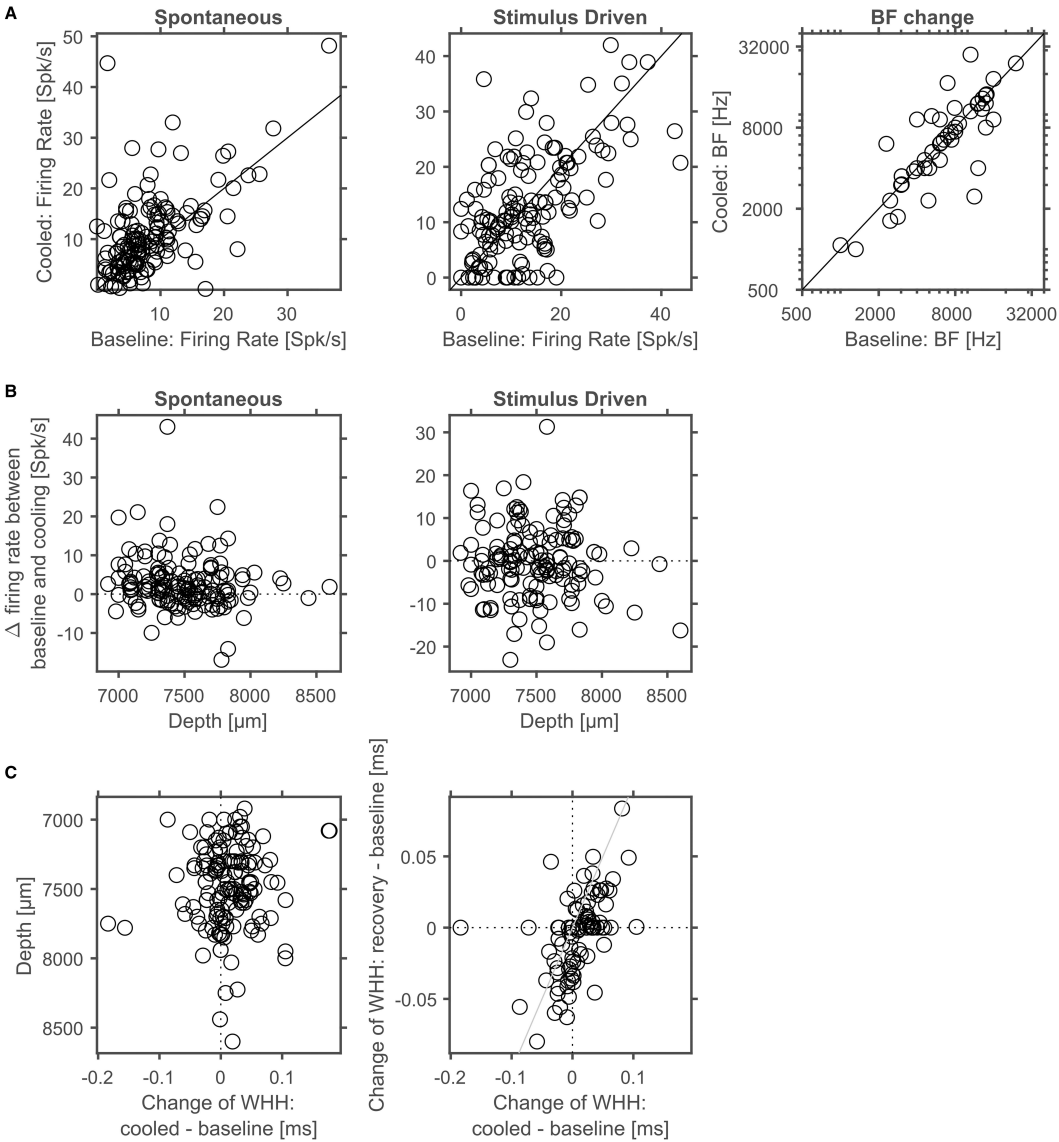
Corticofugal feedback alters spontaneous and stimulus driven activity in thalamus. **(A)** Cortical cooling led to significant modulation of thalamic neurons as indicated by increased spontaneous activity. In contrast, stimulus evoked activity shifted in both directions. The BF of the majority of neurons did not change in response to cooling. **(B)** In thalamus no depth dependent changes of spontaneous or stimulus driven firing rates were observed. **(C)** Changes of spike waveforms in relation to changes observed at different cortical depths were small (< 0.1 ms). In addition, changes in WHH did not recover indicating that at larger distances from the probe observed waveform changes were not related to cooling.

To address whether and how the manipulation of corticofugal feedback *via* cortical cooling affects the physiology of individual neurons in the auditory thalamus, we recorded single units in the MGB under baseline and cooled conditions. First, we explored how spontaneous and stimulus driven firing rates as well as basic frequency tuning were affected by cortical cooling (Figure 9A). For all 3 parameters, bidirectional changes were observed: both spontaneous as well as stimulus driven firing rates could decrease or increase during cooling, while a single unit's best frequency could increase or decrease. Twenty-five out of 52 units tested displayed the same best frequency during baseline and cooling, while for the remaining units the median change was −0.2 octaves (median absolute change = 0.5 octaves). Overall there was a significant relationship between the best frequencies recorded during baseline and cooling (Pearson correlation; Rho = 0.74, *p* = 1.9e-5). During cortical cooling, spontaneous firing rates were significantly increased (Wilcoxon signed rank test, *n* = 144, *Z* = 4.88, *p* = 1.06e-5; 13% of neurons decreased [with a modulation index (MI) > 0.2] while 36% increased their firing rate [MI < −0.2]). In contrast no significant change in stimulus driven firing rates was observed (Wilcoxon signed rank test, *n* = 144, *Z* = −0.47, *p* = 0.87; 28% of neurons decreased [with a modulation index (MI) > 0.2] while 22% increased their firing rate [MI < −0.2]). To address the possibility of direct cooling effects in thalamus, we plotted the changes of spontaneous and stimulus driven firing rates as a function of recording depth. Here, no relationship between depth and single unit firing rates was observed (Pearson correlation; spontaneous activity, Rho = −0.140, *p* = 0.088, stimulus driven, Rho = −0.057, *p* = 0.50, Figure 9B). Changes in shape of spike waveforms were small for thalamic neurons (Figure 9C) and were bidirectional, demonstrating both widening as well as narrowing of spike waveforms, and generally less than ± 0.1 ms (median change = 0.008 ms). Further, no relationship between waveform changes and recording depth was observed (Pearson correlation; Rho = −0.08, *p* = 0.37). To test whether these changes were due to cortical cooling or due to additional factors, we calculated a correlation between changes in spike waveforms contrasting cooled and baseline condition as well as changes during recovery and baseline. Any temperature related changes should be temporary and recover when cooling ceases especially if the temperature change is small given the distance to the cooling probe as in the case of MGB neurons. In contrast, changes in spike waveforms did not recover and consequently were highly correlated between the contrasted conditions (Pearson correlation, *n* = 89, Rho = 0.50, *p* = 6.2e-7). This observation is consistent with small electrode drift during the recording. Therefore, together, the data indicate that cortical cooling does not exert a direct influence on the physiology of neurons in the MGB.

### Effects of Cortical Cooling on MGB Responses to Amplitude Modulated Sounds

**Figure 10:**
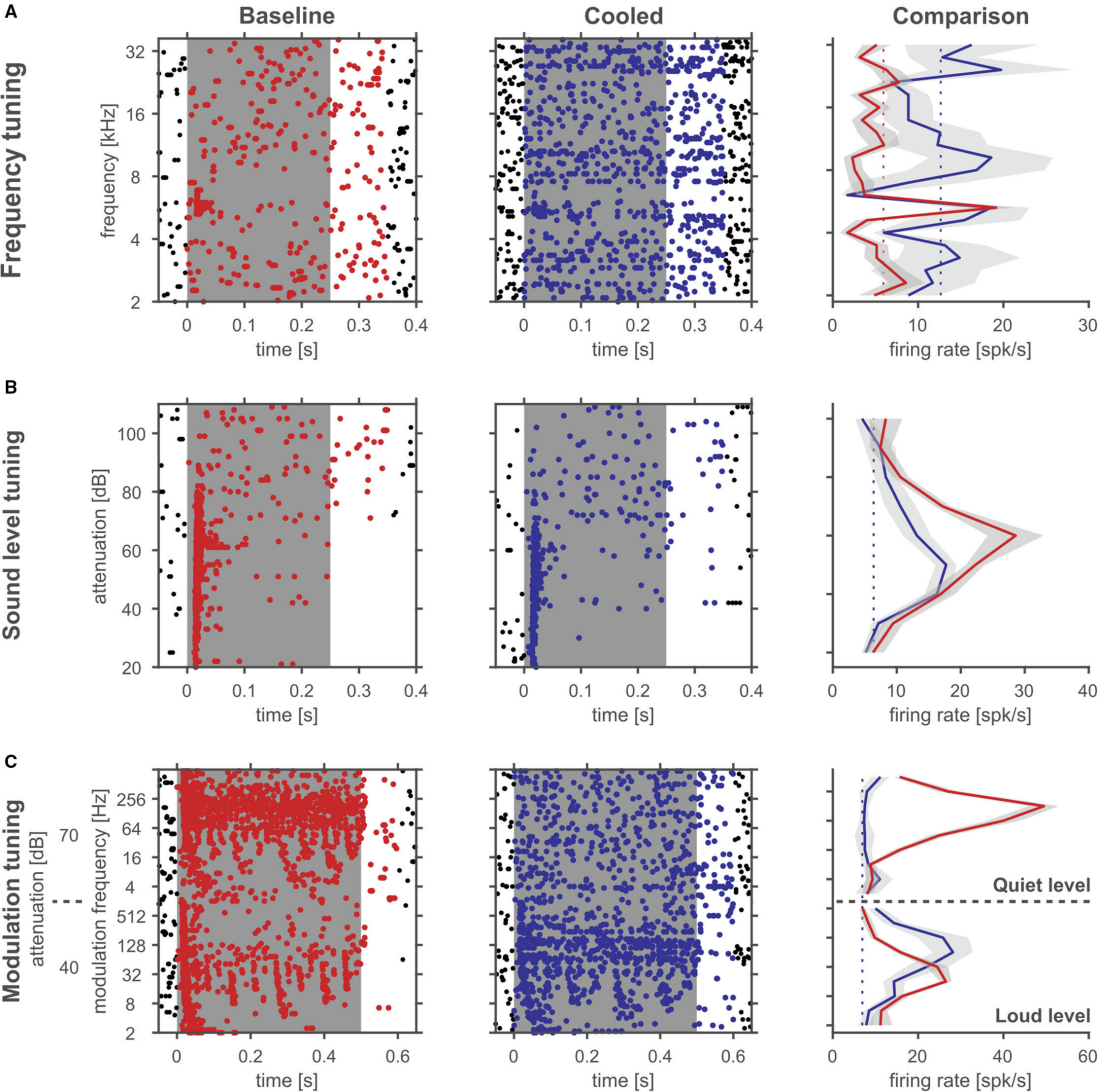
Cortical cooling can lead to large changes in rate level functions and temporal response patterns despite stable frequency tuning. Rasterplots of an example single unit in the auditory thalamus at baseline (left panels) and cooling condition (right panels). The gray shaded area corresponds to the stimulus duration. Stimulus related firing was evaluated based on a time window which included the stimulus duration plus 50 ms. Spikes that contributed to this stimulus related firing were plotted in red during baseline and blue during cooling. For a quantitative comparison average stimulus related firing rates were plotted on the right (gray shadows correspond to the standard error of the mean, red and blue solid lines indicate firing rate during baseline and cooling, respectively, while dashed lines indicate the spontaneous firing rate). **(A)** Responses to pure tones of various frequencies at a fixed sound level were used to determine the neurons' frequency tuning and best frequency (4.7 kHz). **(B)** At the neurons' best frequency pure tones with different sound levels revealed response threshold and a non-monotonic sound level tuning with a best level of 35 dB SPL or 45 dB SPL and a threshold of 25 dB SPL or 35 dB SPL during baseline and in the cooled condition, respectively, and in response to amplitude modulated tones of systematically varied modulation frequency with the best frequency as the carrier frequency presented at threshold and 30 dB above **(C)** during baseline and cooled conditions.

**Figure 11:**
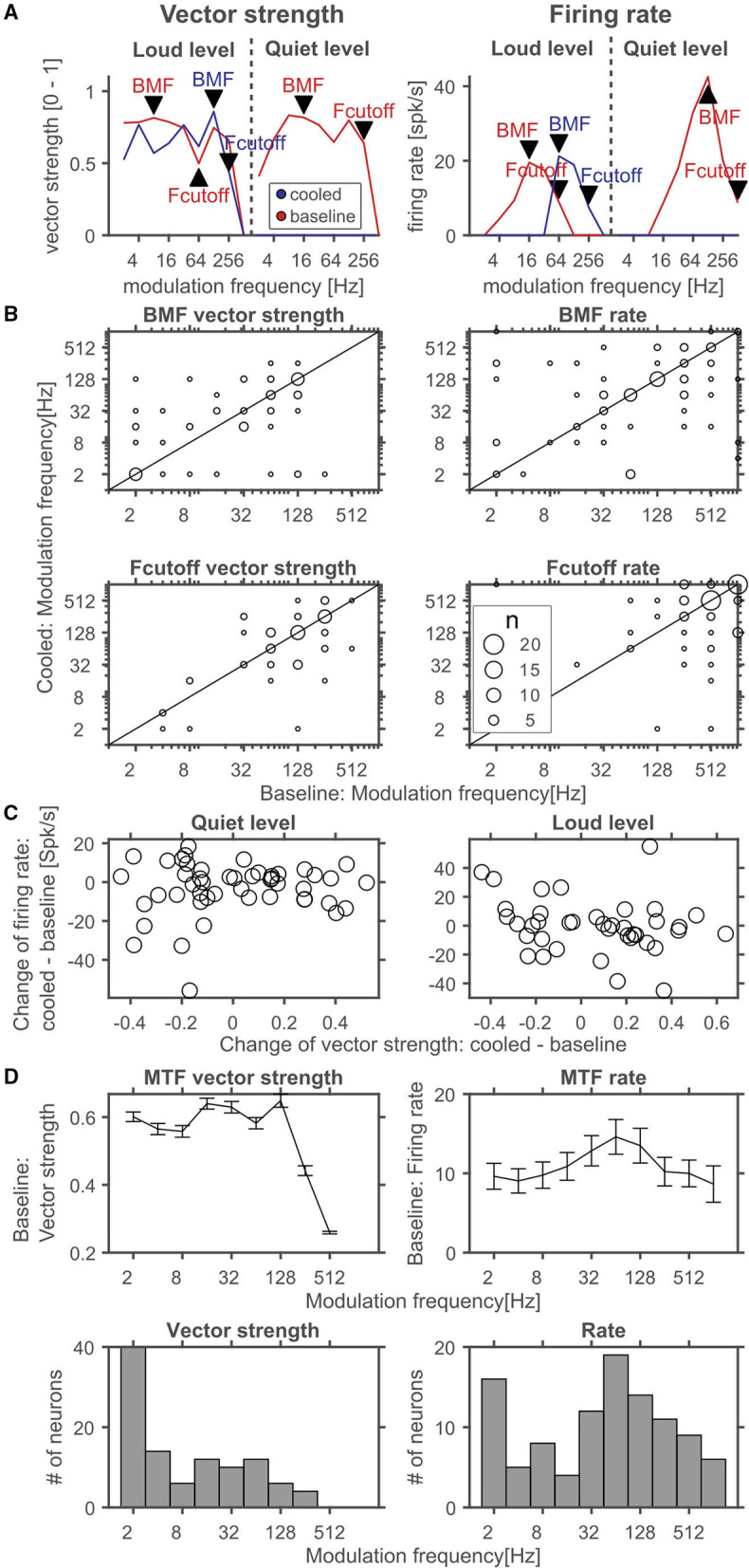
Quantitative analysis of corticofugal influences on thalamic modulation tuning. **(A)** Schematic representation of analyzed parameters in comparison of cooled and baseline conditions. Modulation transfer functions (MTF) based on the vector strength (left panel) during baseline (red) and cooling (blue) were used to identify the best modulation frequency (BMF vector strength) defined as the modulation frequency leading to the highest significant vector strength and a significant response based on the firing rate. The highest frequency leading to a significant vector strength and significant response was taken as the synchronization boundary (Fcutoff vector strength). Similarly, MTFs based on the stimulus related firing rate (right panel) were analyzed to reveal the best modulation frequency (BMF rate) defined as the modulation frequency leading to the highest significant firing rate. The highest modulation frequency leading to a significant response was taken as the rate boundary (Fcutoff rate). Note, that the MTFs shown in **(A)** correspond to the exemplar unit illustrated in Figure 10. The best modulation frequencies and modulation frequency boundaries during baseline and cooling were identified by labeled arrowheads (red – baseline, blue – cooled). **(B)** Scatter plots of best modulation frequencies (BMF) as well as synchronization and rate boundaries (Fcutoff) contrasting cooling and baseline conditions. The sizes of the circles correspond to the number of observations (common legend in the lower right panel). BMFs and Fcutoffs of thalamic neurons were found to change toward lower or higher modulation frequencies with cortical cooling based on vector strength or firing rate. **(C)** Changes in vector strength could in principle be linked to changes in firing rate. However, neither close to the threshold of a neuron (quiet level) nor ca. 30 dB above (loud level) significant correlations were observed. **(D)** During baseline, average vector strength based MTFs calculated from the population of neurons were flat up to 128 Hz modulation and rolled off steeply at higher modulation frequencies (top left panel). Despite this, strongest changes in vector strength observed during cooling occurred at low modulation frequencies for a large number of neurons as indicated by the histogram of largest changes in vector strength as a function of modulation frequency (bottom left panel). Mean MTFs based on the firing rate (top right panel) exhibited a slight peak at 64 Hz modulation and rolled of softly toward slower and faster modulation frequencies. The histogram of neuron counts that had their largest change in firing rate between baseline and cooling also had a peak at 64 Hz modulation and rolled off toward lower and higher modulation frequencies (bottom right panel).

During processing of amplitude modulations, responses in the inferior colliculus are generally synchronized to the sound and thus provide temporally modulated input to the thalamus. Thus, the timing of cortical feedback on thalamic single neurons might influence their processing of temporal modulations. Consequently, we studied how the processing of amplitude modulated sounds might be altered during cortical cooling. After establishing a neuron's best frequency (BF, Figure 10A) and rate-level response function at BF (Figure 10B), amplitude modulated pure tones centered at the BF were presented at 2 different sound levels: close to threshold (0–10 dB above) and 30–40 dB above threshold (Figure 10C). Both, before and during cooling the example neuron depicted in Figure 10 had a BF of 4.7 kHz. However, upon cooling the BF threshold increased to 35 dB SPL from 25 dB SPL during baseline. Further, the response pattern to pure tones remained stable with a phasic increase in firing followed by a tonic suppression. In contrast, responses to AMs at the BF changed substantially. During baseline conditions, the neuron showed clear phase locking to the amplitude modulation up to 32 Hz. For AMs close to threshold the response regime also included non-synchronized responses to faster modulations while 30 dB above threshold only onset responses were observed. During cortical cooling, responses to 25 dB SPL AMs ceased as expected on the basis of the observed rate-level response functions. However, at 55 dB SPL responses to AMs were less synchronized and included a non-synchronized region from 128 Hz modulation frequency which was not observed during baseline. These exemplary data suggest that corticofugal feedback can have a profound effect on the processing of temporal modulations despite stable frequency tuning and response regimes. For quantitative analysis we calculated best modulation frequencies (BMF) and cutoff frequencies (Fcutoff) based on the vector strength (Goldberg and Brown, 1969; Bartlett and Wang, 2007) as a measure of the temporal reliability of responses and based on the firing rate (Figure 11A). Cortical cooling did not just lead to shifts in BMF_vector strength_ toward lower modulation frequencies but also toward higher modulation frequencies. When BMF was calculated based on firing rate (BMF_rate_) a similar pattern emerged in that bidirectional shifts toward higher and lower modulation frequencies were found. For both parameters, BMF_vector strength_ and BMF_rate_, overall no differences between baseline and cooled conditions were observed (Wilcoxon signed rank test, BMF_vector strength_: *n* = 59, *Z* = 0.0378, *p* = 0.969, 31% of neurons with decreased and 32% with increased BMF_vector strength_; BMF_rate_: *n* = 82, *Z* = −0.372, *p* = 0.71, 30% of neurons with decreased and 26% with increased BMF_rate_) but significant correlations between values observed during baseline and cooled conditions (Pearson correlation; BMF_vector strength_: *n* = 59, Rho = 0.40, *p* = 0.001; BMF_rate_: *n* = 82, Rho = 0.41, *p* = 0.0001). The highest frequencies at which synchronized or significant firing rates were observed (Fcutoff_vector strength_ and Fcutoff_rate_, respectively), were also compared between baseline and cooling. As for BMF, bidirectional changes toward lower but also higher modulation frequencies were observed while on average baseline and cooled conditions did not differ (Wilcoxon signed rank test, Fcutoff_vector strength_: *n* = 59, *Z* = 0.255, *p* = 0.80, 27% of neurons with decreased and 25% with increased Fcutoff_vector strength_; Fcutoff_rate_: *n* = 82, *Z* = −0.712, *p* = 0.48, 26% of neurons with decreased and 21% with increased Fcutoff_vector strength_). Here, also significant correlations between conditions were found (Pearson correlation; Fcutoff_vector strength_: *n* = 59, Rho = 0.52, *p* = 2.1e-5; Fcutoff_rate_: *n* = 82, Rho = 0.457, *p* = 1.6e-5). Changes in synchronization of responses to amplitude modulations as studied by the vector strength could in principle be due to concomitant changes in firing rate (Goldberg and Brown, 1969). Therefore, we tested whether changes in vector strength were related to changes in firing rate between baseline and cooling separated for AMs close to threshold and 30–40 dB above (Figure 11C). No significant correlation was observed. In addition, we studied at which modulation frequencies the largest changes in vector strength or firing rate occurred (Figure 11D). Under baseline conditions the averaged modulation transfer function (MTF) calculated for the vector strength was essentially flat until 128 Hz modulation. Despite this, the majority of neurons were found to change strongest at the lowest modulation frequency tested (2 Hz). In contrast, the MTF based on the neurons, firing rates and the histogram of largest changes closely mimicked each other with a peak of the MTF and histogram at 64 Hz.

### Tuning of MGB Neurons to Spatial Locations During Cortical Cooling

**Figure 12:**
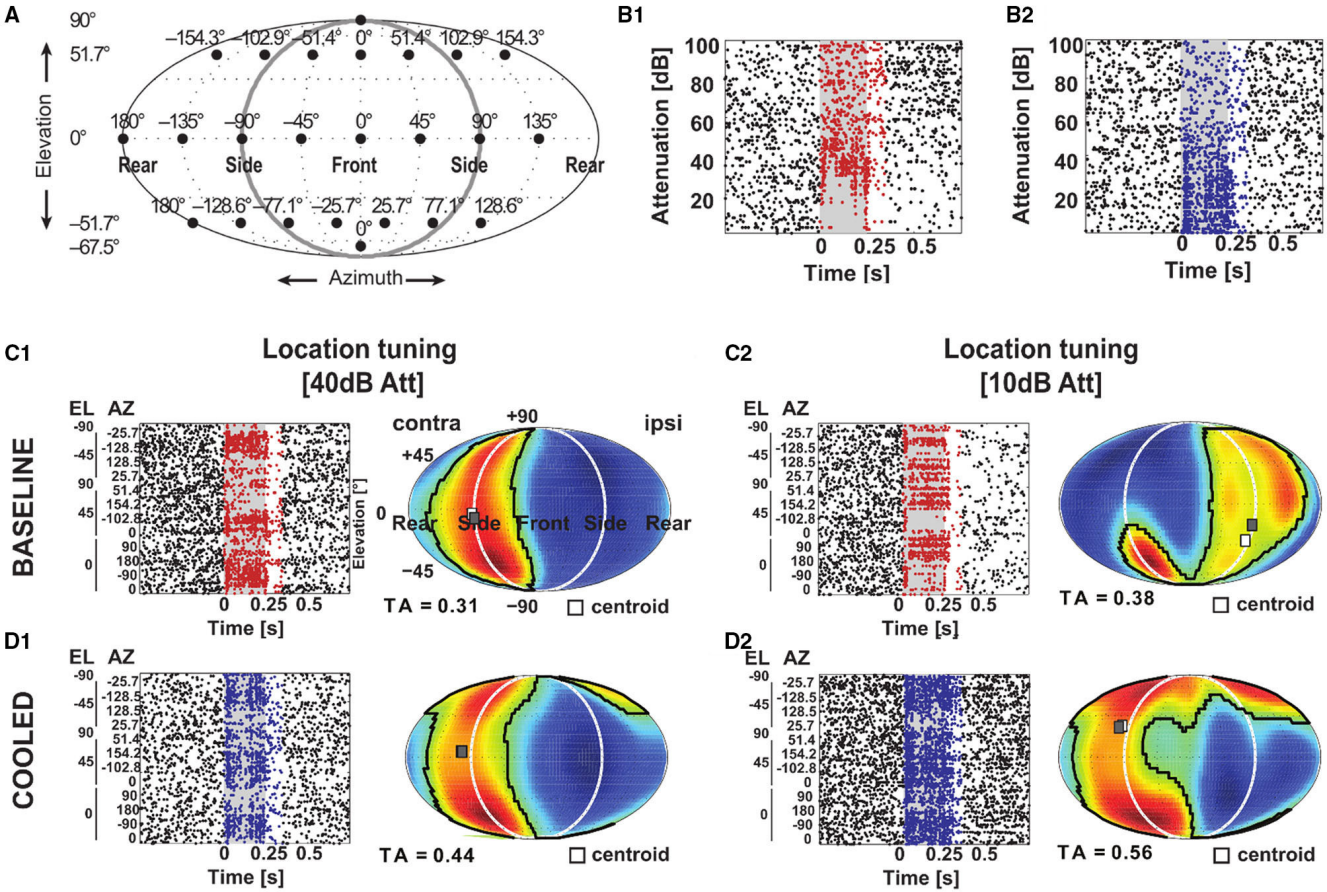
Corticofugal feedback shapes tuning to spatial location in the MGB. **(A)** Schematic of speaker layout to investigate tuning to spatial location. A total of 24 speakers (filled black circles) were positioned at the azimuth and elevation indicated above the speaker and on the left, respectively. The setup was the same as in Remington and Wang (2019). For the rasterplots in **(B–D)** the gray shaded area corresponds to the stimulus duration. Stimulus related firing was evaluated based on a time window which included the stimulus duration plus 50 ms. Spikes that contributed to this stimulus related firing were plotted in red during baseline and blue during cooling. **(B)** Responses to different sound levels of an exemplar neuron under baseline **(B1)** and cooled **(B2)** conditions obtained from the best speaker location identified during baseline 10 dB above threshold. During cooling the sound level tuning turned from non-monotonic to monotonic. **(C,D)** Based on the stimulus related firing rate, a graphical representation of the spatial receptive field was created by projecting the responses at the various speaker locations onto an array of virtual locations using a weighted sum of responses at all speaker locations. These responses to virtual locations expressed in a contralateral-to-ipsilateral axis were plotted as a heatmap using a Fournier projection. Here, warm colors correspond to higher and cold colors to lower firing rates, respectively. The area of responses at least 50% of the mid-point between the maximum and minimum firing rate was taken as the tuning area (TA). The centroid (white square) describes the center of mass of the spatial receptive field defined by the firing rate. During baseline condition, the neuron illustrated in **(B)** mostly responded to speaker locations contralateral to the recorded hemisphere **(C1)** at 10 dB above threshold (see **B1**) but had an ipsilateral receptive field at 30 dB above threshold **(C2)**. **(D)** During cooling, the receptive field remained contralateral 10 dB above threshold **(D1)** but shifted toward the contralateral side 30 dB above threshold **(D2)**.

**Figure 13:**
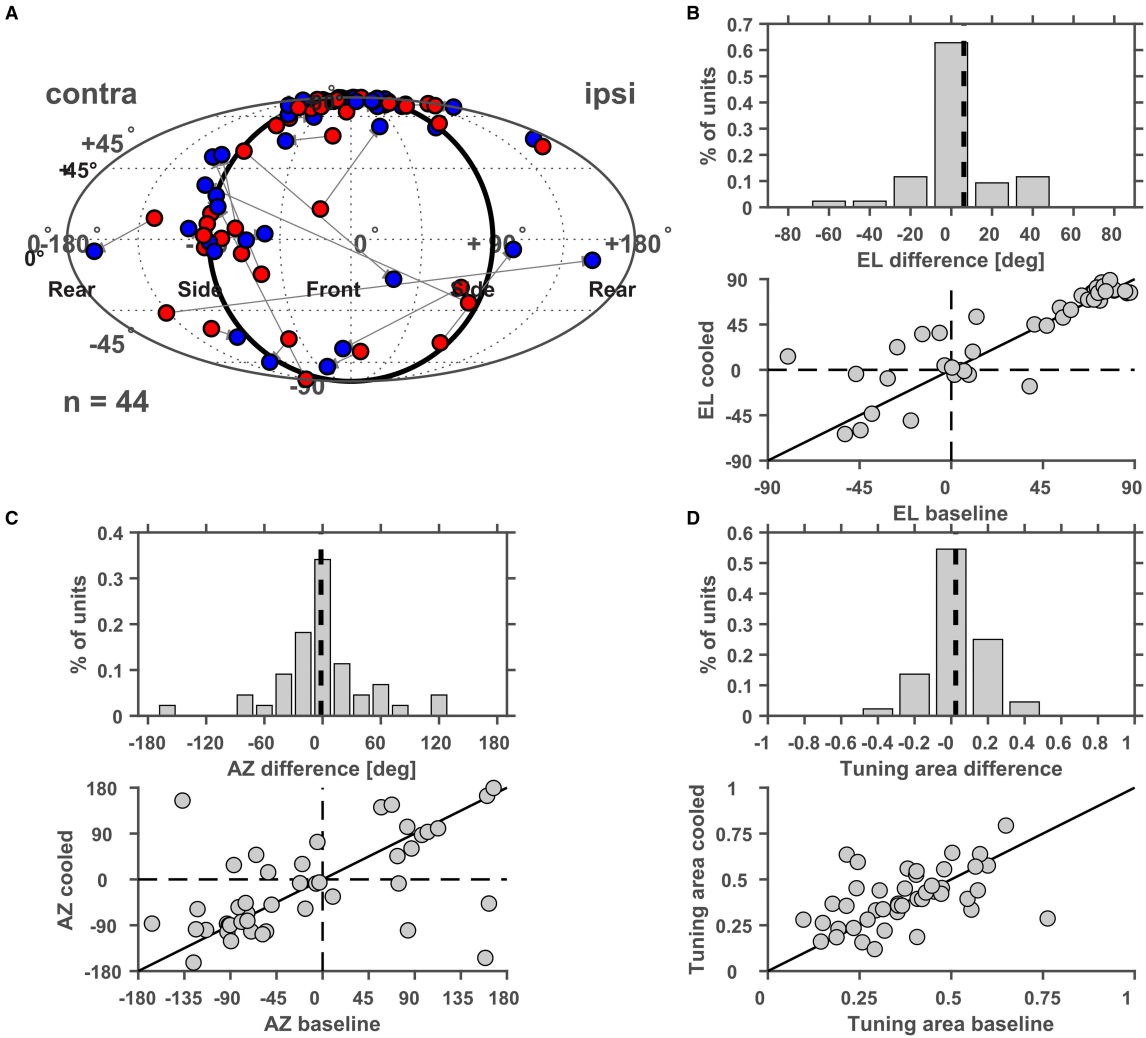
**(A)** Changes of the centroid of the spatial receptive field of thalamic neurons during cortical cooling. The centroid determined under baseline conditions was plotted in red and the centroid under cooled conditions was plotted in blue. The centroids are characterized by their azimuth and elevation and cooling related changes of both parameters were analyzed separately and plotted either as a distribution of changes between cooled and baseline conditions or as a scatter plot of baseline and cooled values. For elevation a decrease or increase was indicated by negative and positive elevation difference values, respectively **(B)**, while azimuth shifts toward the contralateral or ipsilateral side were indicated by negative and positive values, respectively **(C)**. For both parameters the distribution of changes was centered on zero. No significant correlation between azimuth or elevation values recorded during baseline or cooling was observed. **(D)** Changes in the size of the spatial receptive fields as measured by the tuning area were plotted and were again represented either as a distribution (negative values indicate reduced receptive field size under cooled conditions) or as a scatter plot.

Furthermore, we studied cortical influences on processing of spatial location in the MGB. Toward this, neurons were tested with white noise bursts presented from speakers distributed on a sphere of 1 m diameter centered on the animals' head (Figure 12A). Under baseline condition the exemplar neuron in Figure 12 responded in a phasic-tonic manner to noise bursts (Figure 12B1) and displayed contralateral (i.e., responded to sounds from locations opposite from the recorded hemisphere) tuning for spatial location close to threshold but ipsilateral (i.e., responded to sounds from locations at the same side as the recorded hemisphere) tuning at 30 dB above threshold (Figure 12C1). This ipsilateral shift at higher sound levels was accompanied by a suppression of tonic response components at previously driven locations (Figure 12C2). In line with this observation, the rate level response function at the best speaker location was found to be strongly non-monotonic (Figure 12B1). During cooling the pattern of tuning changed quite dramatically. While close to threshold contralateral tuning was still observed (Figure 12D1), the tuning for spatial location changed from ipsilateral to contralateral at 30 dB above threshold (Figure 12D2). In a cooled state some additional responses were also observed: the single unit now also responded to locations ipsilateral and above the median plane (45 degree elevation and 51.4 degree azimuth; Figures 12D1,D2). The rate level response also switched its behavior from non-monotonic to monotonic but exhibited a stable response threshold of 40 dB attenuation (Figure 12B2). As expected the cooling-induced unmasking of previously unseen responses resulted in the receptive field becoming larger (as indicated by the tuning area [TA], see Materials and Methods; from 0.31 and 0.38 TA at threshold and 30 dB above during baseline to 0.44 and 0.56 TA during cooling). For all single units investigated, the centroid (the geometric center of the receptive field calculated *via* the weighted mean of firing rates) of the spatial receptive field was calculated and compared between baseline and cooling conditions (Figure 13A). Although shifts of centroids were observed for a substantial number of units, these shifts were found in random directions (to higher and lower elevations, toward ipsilateral and contralateral azimuths) and did not display a bias toward a particular location, e.g., the contralateral pole. We also split the change in centroid location with respect to elevation (Figure 13B) and azimuth (Figure 13C). In both cases, the distribution of the difference in elevation or azimuth between baseline and cooling was centered on zero (elevation: mean change = 6.1° [upwards]; azimuth: mean change = −1.6° [contralateral]), indicating stable tuning for spatial location in the population (Wilcoxon signed rank test, elevation: *n* = 44, Z = 1.494, *p* = 0.14; azimuth: *n* = 44, Z = 0.292, *p* = 0.77) while individual neurons shifted their location preference. When plotting the azimuth or elevation preference for baseline and cooled conditions, significant correlations between both states were observed (Pearson correlation; elevation: *n* = 44, Rho = 0.87, *p* = 1.5e-14; azimuth: *n* = 44, Rho = 0.533, *p* = 0.0002). The distribution of the change in size of the spatial receptive fields as calculated by TA was slightly skewed toward larger TA during cooled conditions (mean change = 0.024) also suggesting stable receptive field sizes in the population of neurons (Wilcoxon signed rank test, *n* = 44, *Z* = 1.266, *p* = 0.21). The TA observed during baseline and cooled conditions were significantly correlated (Pearson correlation; *n* = 44, Rho = 0.50, *p* = 0.0006).

## Discussion

To study the role of corticothalamic feedback on the processing of sounds in the medial geniculate body of awake, non-human primates we developed a small, freely repositionable cooling probe and compared responses to sounds under conditions of normal and reduced cortical temperature. In cortical neurons we observed cooling-induced increases in the width of the extracellularly recorded spike waveform over distances to the cooling probe of several hundred micrometers. Concomitantly, cortical neurons displayed reduced spontaneous as well as stimulus driven firing rates. At the thalamic level, cortical cooling led to increased spontaneous firing and both increased and decreased stimulus driven activity. Also, response tuning to modulation frequencies of amplitude-modulated tones and spatial tuning to sound source location could be altered in a bidirectional fashion by cortical cooling. Specifically, best modulation frequencies of individual MBG neurons could shift to either higher or lower frequencies based on vector strength or firing rate. Spatial tuning could sharpen or widen, elevation preference could shift toward higher or lower elevations and azimuth tuning could move toward ipsilateral or contralateral locations.

### Cortical Cooling Effects

When a recorded neuron continued to fire during cortical cooling, changes in spike waveforms associated with an increased width at half height were the most obvious effect on the physiology of individual neurons. Similar effects have been reported *in vitro* (Volgushev et al., 2000a,b) and *in vivo* in the visual cortex of anesthetized cats (Girardin and Martin, 2009). Such widening of spike waveforms was observed at a distance of at least 2 mm underneath the cooling probe, demonstrating that the local temperature was lowered throughout all cortical layers in the auditory cortex (with an estimated thickness of 1.5–2 mm; Aitkin et al., 1986) including the corticofugal output layers 5 and 6 (Llano and Sherman, 2009). The correlation of changes in spike width with distance from the cooling probe allows to disentangle direct (i.e., temperature related) and indirect (i.e., due to changes in network activity) effects of cooling as suggested before (Girardin and Martin, 2009). Interestingly, even when large changes in spike width were observed neuronal activity was not always suppressed but could also be enhanced. While with our data it is not possible to relate those changes to a particular mechanism, they can be explained by the differential effects of lowering temperature on membrane potential, spike threshold, transmitter release and synaptic transmission (Volgushev et al., 2000a,b, 2004). In these experiments complete cessation of neuronal firing was only observed upon lowering the local temperature to below 10°C. As demonstrated in our experiments and others this is not observed for distances from the cooling probe larger than 1 mm (Lomber et al., 1999; Coomber et al., 2011) even when the dura temperature is lowered to 1–5°C. Therefore, at least in unanesthetized animals, the effect of cooling even on relatively local network activity seems to be more diverse than previously suggested based on experiments in anesthetized preparations (Villa et al., 1991; Lomber et al., 1999; Volgushev et al., 2000b). On average, though, cortical cooling leads to a reduction of activity up to a distance of at least 2 mm. This suggests that the main effect of superficial cooling on corticofugal feedback originating from layers 5 and 6 is a reduction but not a cessation of feedback. This potentially limited reduction of corticothalamic feedback can nonetheless have strong network effects as shown by experiments with moderate cooling in the barrel cortex of rats (Burkhanova et al., 2020).

### Effects of Cortical Feedback on Thalamic Processing of Temporally Modulated Sounds

The control of thalamocortical information flow *via* corticofugal feedback (Ibrahim et al., 2021; Saldeitis et al., 2021) can be expected to have strong influence on cortical responses despite small changes to thalamic population activity (Wang et al., 2010; Ibrahim et al., 2021). This can be explained by the convergence of synchronized, weak thalamocortical inputs (Bruno and Sakmann, 2006) in combination with desynchronized thalamic neurons (Wang et al., 2010; Ibrahim et al., 2021) or reduced stimulus locked firing due to switches of thalamic response mode (Whitmire et al., 2021) under altered corticothalamic and/or corticofugal feedback.

Corticofugal feedback arises from layers 5 and 6 with different projection patterns and synaptic strengths (Llano and Sherman, 2009). In the rodent, A1 to MGBv feedback is predominantly of the so-called modulator type (i.e. small synapses) (Bartlett et al., 2000) while non-lemniscal subdivisions receive more driver type projections (large synapses) (Llano and Sherman, 2008). As our experiments did not distinguish between subnuclei of the MGB our work could not disentangle the relative changes within the various subdivisions. Corticothalamic connections outnumber the thalamic inputs and dominate the synaptic background in thalamic neurons and thereby determine the thalamocortical transmission (Wolfart et al., 2005). For example, in mice moderate cooling (16–21°C) eliminates silent states in the somatosensory thalamus (Sheroziya and Timofeev, 2015). Silencing auditory cortex optogenetically leaves the MGB less excitable, with higher reliability and linearity but keeps general tuning stable (Lohse et al., 2020). Therefore, it is expected that not all aspects of thalamic sound processing can be influenced by cortex. In the current study with awake non-human primates we have documented that two important aspects of auditory processing, namely processing of spatial location for orientation to sounds and temporally modulated sounds relevant for example for speech comprehension (Ding et al., 2017), can be modulated by cortical control.

The majority of thalamic neurons respond in a synchronized manner at least to some temporal modulation frequencies (Bartlett and Wang, 2007, 2011). Former work has demonstrated that thalamocortical loops are involved in rhythmic activity (Llinás et al., 2005). Corticothalamic neurons have likewise been shown to affect fast-spiking interneurons in the cortex to directly reset slow rhythmic activity but not by changing activity of thalamic resetter neurons (Guo et al., 2017). Thus, corticothalamic feedback plays a decisive role in oscillatory activity. Therefore, we reasoned that manipulating precisely timed cortical feedback would influence thalamic processing of temporal modulations. Interestingly, while changes in temporal precision of responses were bidirectional, the largest changes were observed for low modulation frequencies in line with the abovementioned literature.

### Effects of Cortical Feedback on Thalamic Processing of Spatial Locations

Our data are the first to describe tuning to spatial location in the MGB collected from awake common marmosets. All recordings were performed in the same setting described in an earlier study which investigated the representation of the full spatial field in the auditory cortex (Remington and Wang, 2019). While a detailed description of spatial receptive field (SRF) properties is outside of the scope of the current study, some comparisons seem noteworthy: in general thalamic SRFs were mostly larger than their counterparts in the auditory cortex with tuning areas of 0.36 ± 0.15 (mean ± STD) in the MGB and tuning areas of 0.14, 0.24, and 0.34 in field CM/CL, A1 and R/RT, respectively (Remington and Wang, 2019). Similar to observations in the auditory cortex the SRF of thalamic neurons could increase or shrink with increasing sound level. At the population level however, spatial selectivity was found to be level tolerant both for the auditory cortex (Remington and Wang, 2019) and the MGB.

In our experiments, we have further documented changes in tuning to spatial location in the MGB during altered cortical feedback. Processing of spatial location is based on three cues: interaural level and time differences as well as head related directional filtering (Grothe et al., 2010). While level and time differences are processed in brainstem nuclei, the inferior colliculus has been argued as being the first stage in which all three cues are combined (Slee and Young, 2011). Further, corticofugal modulation of sound processing or input selection of relevant stimuli or sound features is not only possible in the auditory thalamus (Guo et al., 2017) but has been shown throughout the auditory pathway (Malmierca et al., 2015; Suga, 2020; Asilador and Llano, 2021) including the cochlear nucleus (Luo et al., 2008) and the inferior colliculus (Nakamoto et al., 2008; Straka et al., 2015; Qi et al., 2020). Cooling of auditory cortex shifts the sensitivity of inferior colliculus neurons to interaural level cues (Nakamoto et al., 2008). However, corticothalamic and corticocollicular feedback arises from layer 6 or 5, respectively (Malmierca et al., 2015) and our data suggest that both cortical output layers are affected by cortical cooling. Therefore, it would be interesting to investigate the relative contributions of corticothalamic feedback vs. the possibly altered colliculothalamic input due to corticocollicular feedback.

### Limitations of the Current Study

In our study we have used cooling to manipulate corticofugal feedback. As a technique cooling has its advantages in being flexible and quickly reversible. However, disadvantages include the relatively large affected region which is difficult to control and the lack of temporal specificity which might play a role especially during processing of temporally dynamic stimuli. The former point essentially precluded to investigate the relationship between the tonotopic position in AI being cooled and the tonotopic position in MGB being affected (see e.g., Suga, 2020) while the latter prevented an analysis of a potential time dependence of corticofugal feedback on thalamic processing.

Although the cortico-thalamic connection contains both excitatory (direct) as well as inhibitory influences (indirect *via* the thalamic reticular nucleus) it cannot be ruled out that the bidirectionality of changes we observed is due to the spatially relatively broad and cell type unspecific manipulation invoked *via* cortical cooling. Even the same corticofugal connection can have various effects on the target region based on how it is activated (Vila et al., 2019). Ideally, an optogenetic experiment with anterograde labeling of cortico-thalamic neurons and optical stimulation in the thalamus would be performed (Fenno et al., 2011; Yizhar et al., 2011; Vila et al., 2019; Williamson and Polley, 2019). If successful in primates, optogenetic experiments could selectively target layer 5 or layer 6 projection neurons to investigate their separate roles for adjusting thalamic processing.

Further experiments should delineate the respective role of subdivisions of the MGB as well as the role various circuit elements of the cortico-thalamo-cortical, cortico-colliculo-thalamo-cortical and various other corticofugal feedback loops (Winer, 2005; León et al., 2012; Saldaña, 2015; Lohse et al., 2021), e.g., the thalamic reticular nucleus, the inferior colliculus or even lower brainstem centers play in adjusting thalamic processing in awake primates. Toward this end, future studies should include many more neurons, confirm their anatomical locations and expand observations to the thalamic reticular nucleus as well as the shell and core of the inferior colliculus.

### Future Directions

One function of corticofugal feedback might be to adjust thalamic processing to focus on relevant stimulus features in a given situation-dependent context (Guo et al., 2017). This idea is further supported by findings describing the activation of corticothalamic projection neurons during movement preparation (Clayton et al., 2021). In light of the available literature, our main finding of bidirectional changes for many parameters we investigated, suggests that the thalamus does act like a stimulus filter that can be adjusted according to behavioral needs. Future experiments should study thalamic processing of complex sounds during behavioral tasks and under conditions of manipulated corticofugal feedback in primates to investigate the flexibility of corticofugal influences.

## Data Availability Statement

The raw data supporting the conclusions of this article will be made available by the authors, without undue reservation.

## Ethics Statement

The animal study was reviewed and approved by Institutional Animal Care and Use Committee of the Johns Hopkins University.

## Author Contributions

MJ, FO, and XW contributed to conception of the study, discussed, and interpreted the data. MJ and XW designed the study. MJ performed all experiments, data analysis, and wrote the first draft of the manuscript. FO and XW wrote sections of the manuscript. All authors contributed to manuscript revision, read, and approved the submitted version.

## Funding

This work was supported by the National Institutes of Health grants DC003180 and DC005808 (XW) and a grant from the Deutsche Forschungsgemeinschaft (DFG, SFB-TRR 31, TP A03) to FO. The funders had no role in study design, data collection and analysis, decision to publish, or preparation of the manuscript.

## Conflict of Interest

The authors declare that the research was conducted in the absence of any commercial or financial relationships that could be construed as a potential conflict of interest.

## Publisher's Note

All claims expressed in this article are solely those of the authors and do not necessarily represent those of their affiliated organizations, or those of the publisher, the editors and the reviewers. Any product that may be evaluated in this article, or claim that may be made by its manufacturer, is not guaranteed or endorsed by the publisher.
